# Traditional Uses, Phytochemicals, Biological Activities, and Biotechnological Applications of *Serjania* Species: A Review of Current Knowledge and Future Prospects

**DOI:** 10.3390/molecules31091477

**Published:** 2026-04-29

**Authors:** Ana Belem Rubio-García, Cecilia Guadalupe de Loza-García, Jorge Manuel Silva-Jara, Napoleón González-Silva, Luis Antonio Ramirez-Contreras, Zuamí Villagran, Omar Graciano-Machuca, Jessica del Pilar Ramírez-Anaya, Fernando Martínez-Esquivias, Luis Miguel Anaya-Esparza

**Affiliations:** 1 Centro Universitario de Los Altos, Universidad de Guadalajara, Tepatitlan de Morelos 47620, Mexico; ana.rubio6413@alumnos.udg.mx (A.B.R.-G.); cecilia.deloza0624@alumnos.udg.mx (C.G.d.L.-G.); napoleon.gonzalez@cualtos.udg.mx (N.G.-S.); luis.ramirez3685@alumnos.udg.mx (L.A.R.-C.); blanca.villagran@academicos.udg.mx (Z.V.); fernando.mesquivias@academicos.udg.mx (F.M.-E.); 2 Centro Universitario de Ciencias Exactas e Ingenierías, Universidad de Guadalajara, Guadalajara 44430, Mexico; jorge.silva@academicos.udg.mx; 3 Centro Universitario de Los Valles, Universidad de Guadalajara, Carretera a Guadalajara Km. 45.5, Ameca 46708, Mexico; omargmachuca@academicos.udg.mx; 4 Departamento de Ciencias Computacionales e Innovación Tecnológica, Centro Universitario del Sur, Universidad de Guadalajara, Av. Enrique Arreola Silva 883, Ciudad Guzmán 49000, Mexico; jessicar@cusur.udg.mx

**Keywords:** therapeutic potential, phytochemicals, saponins, bioactivity, health

## Abstract

The genus *Serjania* (family Sapindaceae) comprises more than 240 species, primarily distributed in Brazil and Mexico, and it exhibits considerable ethnobotanical and therapeutic potential. Ethnobotanical evidence documents the widespread use of decoctions prepared from the leaves, stems, and roots of *Serjania* species for the treatment of gastrointestinal disorders, renal pain, inflammatory conditions, and infections. Among the most extensively studied species are *S. marginata*, *S. erecta*, *S. lethalis*, *S. caracasana*, *S. goniocarpa*, *S. schiedeana*, *S. yucatenensis*, *S. triquetra*, and *S. racemose*. Phytochemical research has identified a diverse array of bioactive secondary metabolites, including saponins, flavonoids, phenolic acids, tannins, and terpenoids. Significant experimental evidence supports the broad spectrum of biological activities of these *Serjania* species, including antimicrobial, anti-inflammatory, antioxidant, gastroprotective, antihypertensive, analgesic, antivenom, cytotoxic, antimutagenic, anti-ulcer, photoprotective, antiparasitic, and vasorelaxant effects, as demonstrated in both in vitro and in vivo models. Although preliminary toxicity assessments of extracts from some *Serjania* species in murine models, *Oreochromis niloticus* (Nile tilapia), and *Artemia salina* suggest a favorable safety profile, significant research gaps remain. Additionally, several *Serjania* species have shown potential as natural pesticides and bioherbicides, highlighting their relevance in agricultural applications. Future studies should prioritize the isolation and structural characterization of individual bioactive compounds, as well as the elucidation of their molecular mechanisms of action, moving beyond crude extract-based screening approaches. Overall, this review summarizes current knowledge on traditional uses, phytochemical composition, biological activities, and biotechnological applications of *Serjania* species.

## 1. Introduction

Traditional/folk medicine, which relies on nature-based remedies, frequently employs plant-based preparations for health maintenance and disease management [1,2]. Since antiquity, plant-derived products have played a central role in diverse traditional medicinal practices for the treatment of non-transmissible chronic diseases (e.g., cancer, diabetes, hypertension, and musculoskeletal disorders), infectious conditions, wound healing, and burn management [3]. Common preparation methods include maceration, decoction, infusion, and poultices from different plant parts such as roots, bark, seeds, peels, and leaves [1]. Within this framework, several species of the genus *Serjania* (family Sapindaceae) have gained increasing scientific attention over the past two decades due to their reported therapeutic properties and bioactive potential [4,5,6,7].

Although approximately 240 *Serjania* species have been documented worldwide, most research has primarily focused on botanical identification. In contrast, ethnobotanical investigations report that decoction prepared from leaves, stems, and roots, particularly of *S. marginata*, *S. erecta*, and *S. lethalis* and in less proportions *S. caracasana*, *S. goniocarpa*, *S. schiedeana*, *S. yucatenensis*, *S. triquetra*, and *S. racemosa* have traditionally been used to treat gastrointestinal disorders, ulcers, bacterial infections, cancer [8,9,10,11,12], inflammatory conditions, dermatological diseases, diarrhea, fever, hypertension [4,12,13,14,15,16,17], renal pain and nephrolithiasis [17,18,19], leg ulcers [20,21,22], vomiting, headache [21], hepatitis, and urinary tract infections [6,7,23].

Phytochemical studies have demonstrated that *Serjania* species contain a diverse range of secondary metabolites, including phenolic acids, flavonoids, tannins, saponins, alkaloids, terpenes, and fatty acids [10,12,13,16,24,25]. These compounds have been associated with multiple biological activities (often in vitro, and in some in vivo studies using murine models), such as antimicrobial [5], anti-inflammatory, antinociceptive [11], antioxidant [13], cytotoxic [26], antimutagenic [8,10], gastroprotective [27], antidiarrhea [10], photoprotective [5], antiparasitic [28], analgesic [4], neuroprotective [14], antihypertensive [15], antivenom [29], and antileishmanial effects [30]. Moreover, several studies suggest promising biotechnological applications, including weed control [19], insecticidal and pesticide activities [31,32], and the isolation of associated microorganisms with biotechnological relevance [33].

Despite this growing evidence, only a limited number of *Serjania* species (approximately eight to ten) have been investigated regarding their ethnobotanical relevance, phytochemical composition, biological activities, or biotechnological uses. Therefore, the present narrative review aims to summarize current knowledge on the ethnobotanical uses, phytochemical profiles, biological activities, and emerging biotechnological applications of *Serjania* species, highlighting research gaps and future perspectives for compound-driven development.

## 2. Literature Search, Selection, and Data Analysis

### Literature Search

This narrative review was conducted using a structured, comprehensive literature search to identify studies on the phytochemistry, biological activities, and traditional uses of *Serjania* species. The literature search was performed in three major electronic databases: PubMed, Scopus, and Web of Science, covering publications from database inception through 17 March 2026. To complement this search and minimize publication bias, an additional manual search was conducted using Google Scholar. The search equation used across databases was as follows: (“*Serjania triquetra*” OR “*Serjania species*” OR “*Serjania*” OR “soapberry vine”) AND (pharmacological OR “biological activity” OR “phytochemical” OR “ethnopharmacology” OR “traditional medicine” OR “bioactive compounds” OR “medicinal plant” OR “antioxidant” OR “anti-inflammatory” OR “antimicrobial” OR “anticancer” OR “hepatoprotective” OR “neuroprotective”). No restrictions were applied regarding publication year, language, or geographic region. Only articles published in peer-reviewed journals were considered. Peer-reviewed journal articles and relevant conference proceedings were considered for inclusion in this review.

The literature search conducted in PubMed, Scopus, and Web of Science yielded 423 records potentially relevant to the scope of this narrative review. After removing duplicate entries and excluding studies that did not meet the predefined thematic focus, a total of 57 publications were retained for further analysis. Therefore, documents included ethnobotanical aspects of *Serjania* species, qualitative and quantitative phytochemical characterization, and examined toxicological profiles. In addition, a substantial proportion of the included studies evaluated the biological activities of *Serjania*-derived extract and fractions using in vitro and in vivo experimental approaches.

The gathered evidence was subsequently examined using a qualitative approach, with particular emphasis on identifying patterns linking phytochemical composition to the observed biological and biotechnological effects.

## 3. Traditional Uses of *Serjania* Species

**Figure 1:**
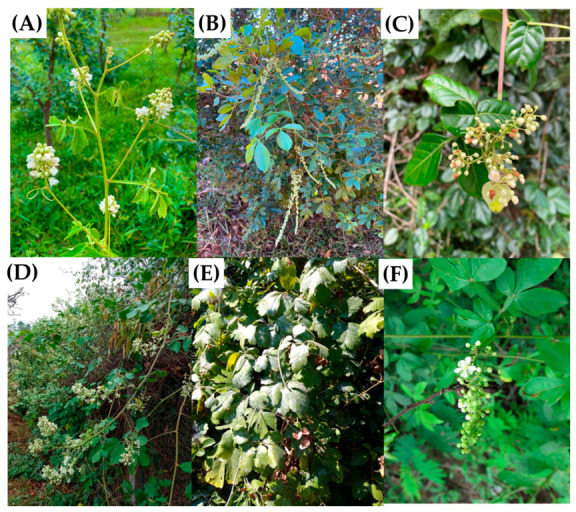
*Serjania* species (**A**) *S. erecta*, (**B**) *S. lethalis*, (**C**) *S. salzmaniana*, (**D**) *S. glabrata*, (**E**) *S. triquetra*, and (**F**) *S. marginata*. The images were obtained from Ínaturalist.org under CC BY and CC BY-NC 4.0 licenses. Detailed information on images and their collaborators is given in Supplementary Information.

The genus *Serjania* belongs to the family Sapindaceae, which comprises trees, shrubs, lianas, and climbing vines predominantly distributed in tropical and subtropical regions worldwide [34]. Native to the Neotropical region, this genus encompasses approximately 240 species, with Brazil representing the primary center of diversity (117 species), followed by Mexico (59 species, 33 of which are endemic) [35]. *Serjania* species are typically characterized as climbing shrubs or woody lianas with brown bark, triquetrous stems, and rhomboidal leaves arranged in clusters [6,36,37]. Figure 1 shows some *Serjania* species.

**Figure 2:**
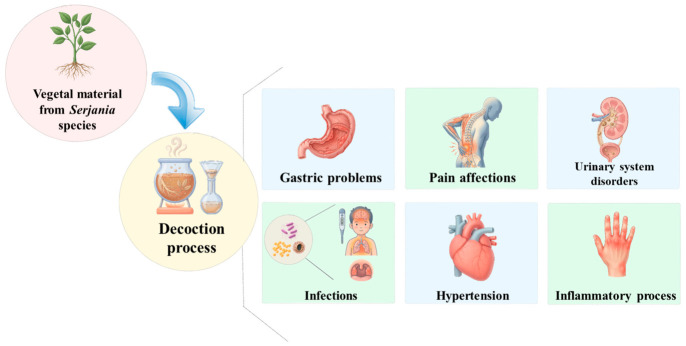
Traditional medical uses of *Serjania* species.

Decoctions of leaves, stems, and roots of different *Serjania* species have been traditionally used for various therapeutic purposes across various regions (Figure 2).

Table 1 summarizes the alleged therapeutic uses of the most extensively studied *Serjania* species and places in which they are mainly used. These decoctions are mainly used for treating gastric problems, pain, infections, hypertension, inflammation, and urinary system disorders, among others.

## 4. Phytochemicals Reported in *Serjania* Species

Phytochemicals comprise a structurally diverse group of naturally occurring compounds, including flavonoids, phenolic acids, terpenes, tannins, alkaloids, and saponins, among others. These compounds are secondary metabolites synthesized via the phenylpropanoid, mevalonate, and shikimate biosynthetic pathways [41]. Many phytochemicals exhibit significant biological activities and have historically served as the basis for traditional therapeutic applications and modern drug discovery efforts [42]. In this context, the documented ethnobotanical uses of *Serjania* species provide a strong rationale for systematic phytochemical investigations [6,43].

Despite the taxonomic diversity of the genus, phytochemical characterization has been conducted in only a limited number of species, including *S. marginata*, *S. erecta*, *S. lethalis*, *S salzmanniana*, *S. caracasana*, *S. goniocarpa*, *S. schiedeana*, *S. yucatanensis*, and *S. triquetra* (Table 2). Among them, saponins, terpenes, and flavonoids represent the predominant classes of compounds. Leaves are the most extensively investigated plant organ, likely reflecting their widespread ethnobotanical use; however, stems and roots have also been investigated (Table 1). Phytochemical identification has primarily been performed using organic solvent extracts (e.g., methanolic, ethanolic, and chloroform) and aqueous preparations. Additionally, certain *Serjania* species have been reported to contain fatty acids and essential oils, further expanding their chemical diversity.

### 4.1. Phenolic Compounds

Phenolic compounds represent one of the most abundant classes of secondary metabolites in higher plants, surpassed only by carbohydrates. Structurally, they range from simple phenols to highly polymerized compounds, including phenolic acids, flavonoids, and proanthocyanidins [52]. These metabolites have been reported in several *Serjania* species (Table 2).

Cinnamic acid derivatives, tannins, flavonoids, and flavonoid glycosides have been identified in the leaves and roots of *S. marginata* [5,10,11,25]. Chromatographic analyses of leaves allowed for the characterization of individual phenolic constituents, including quercetin derivatives [11,44], protocatechuic acid, catechin derivatives, apigenin derivatives, cassiaoccidentalin A–C, luteolin derivatives [25], proanthocyanidin derivatives [44], and glycosylated flavone derivatives [25]. For its part, NMR analysis has identified the presence of flavonoids in *S. racemosa* leaves [12].

In *S. erecta*, qualitative analyses of leaves have revealed flavonoids, tannins [29], glycosylated flavonoids [13], and catechins [16]. The individual phenolic profile includes quercetin, catechin, kaempferol, and apigenin derivatives [4,14,17]. Additionally, flavonoids, tannins, and catechins have been reported in the roots and stems of *S. erecta* [15,16].

In *S. lethalis*, flavonoids and benzoic acid derivatives have been detected in leaves [30,47], whereas quercetin has been identified in the aerial parts of *S. caracasana* [39]. In stems of *S. schiedeana*, total flavonoids, tannins, and epicatechin derivatives have been reported [36,40].

**Figure 3:**
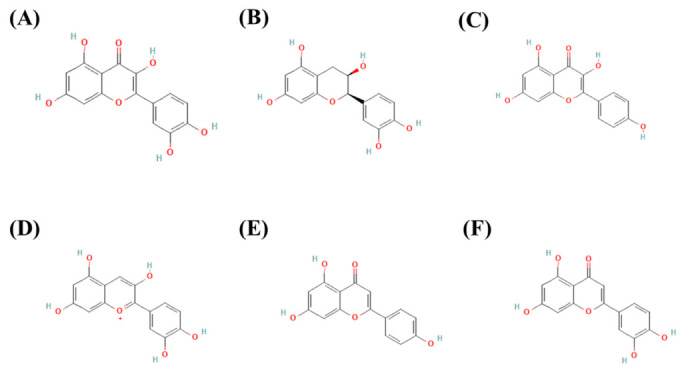
Chemical structures of main phenolic compounds reported in *Serjania* species: (**A**) Quercetin (CID: 5280343), (**B**) Epicatechin (CID: 72278), (**C**) Kaempferol (CID: 5280863), (**D**) Cyanidin (CID: 128861), (**E**) Apigenin (CID: 5280443), and (**F**) Luteolin (CID: 5280445). The chemical structures and their compound identification (CID) were sourced from PubChem [53].

Phenolic compounds are widely recognized for their antioxidant properties and for mitigating oxidative-stress-related chronic degenerative diseases [52]. However, to date, there are no reports on phenolic compounds in *S. goniocarpa*, *S. yucatanensis*, or *S. triquetra*. Figure 3 shows the chemical structures of the most representative phenolic compounds reported in *Serjania* species. These compounds have been identified in other plant species.

### 4.2. Terpenoids

Terpenoids constitute one of the largest and most structurally diverse classes of secondary metabolites. They are biosynthesized from five-carbon isoprene (C_5_H_8_) units and include both volatile and non-volatile compounds [54]. Based on the number of isoprene units, they are classified as monoterpenoids, sesquiterpenoids, diterpenoids, sesterterpenoids, triterpenoids, tetraterpenoids, and polyterpenoids [55]. Due to their lipophilic nature, terpenoids exhibit a broad spectrum of biological activities, including antioxidant, antimicrobial, neuroprotective, and antimalarial effects [55,56]. In this context, various terpenoids have been reported in some *Serjania* species (Table 2).

In *S. erecta*, terpenoids have been qualitatively identified in leaves, shrubs, and stems [16,29,45]. In *S. lethalis* leaves, chromatographic analyses have been identified several individual terpenoids, including α-cubene, 4-epi-cubedol, (-)-spathulenol, caryophyllene oxide, C_14_H_22_O_2_ and (-)-loliolide, 6,10,14-Trymethyl-2-pentadecacone, phytol, (E)-phytol, phytol acetate, 4,8,12,16-tretamethylheptadecan-4-olide, β-amyrone, β-amyrin, lup-20(29)-en-3-one, lup-20(29)-en-3-ol, glutinone, β-amyrin acetate, α-thujene, δ-terpinene, thymol, carvacrol, and β-caryophyllene [20,30].

In aerial parts of *S. caracasana*, spathulenol, 6,10,14-Trimethyl-2-pentadecanone, β-sitosterol, β-amyrin, friedelin, stigmasterol, and β-sitosterol glucoside have been reported [39]. In *S. goniocarpa*, goniocarpic acid and phytol have been identified in leaves [21], whereas lup-20(29)-en-3-one and β-caryophyllene oxide have been reported in *S. yucatanensis* leaves [22].

**Figure 4:**
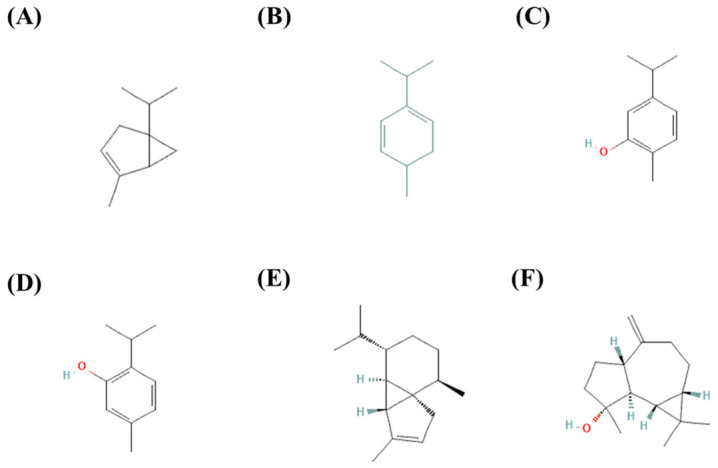
Chemical structures of main terpenoids reported in *Serjania* species: (**A**) α-Thujene (CID: 17868), (**B**) δ-Terpinene (CID: 6428962), (**C**) Carvacrol (CID: 10364), (**D**) Thymol (CID: 6989), (**E**) α-Cubebene (CID: 442359), and (**F**) (-)-Spathulenol (CID: 13854255). The chemical structures and their compound identification (CID) numbers were sourced from PubChem [53].

Additionally, phytol, phytone, 4,8,12,16-tetramethylpentadecan-4-olide, and various fatty acid methyl esters have been identified in stems of *S. schiedeana* [51]. Additionally, stigmasterol, oleanolic acid, morolic acid, hederagenin, and 11α-hydroperoxy-hederagenin have been isolated from leaves of *S. triquetra* [7]. Figure 4 shows the chemical structures of the most representative terpenoids reported in various *Serjania* species.

### 4.3. Saponins

**Figure 5:**
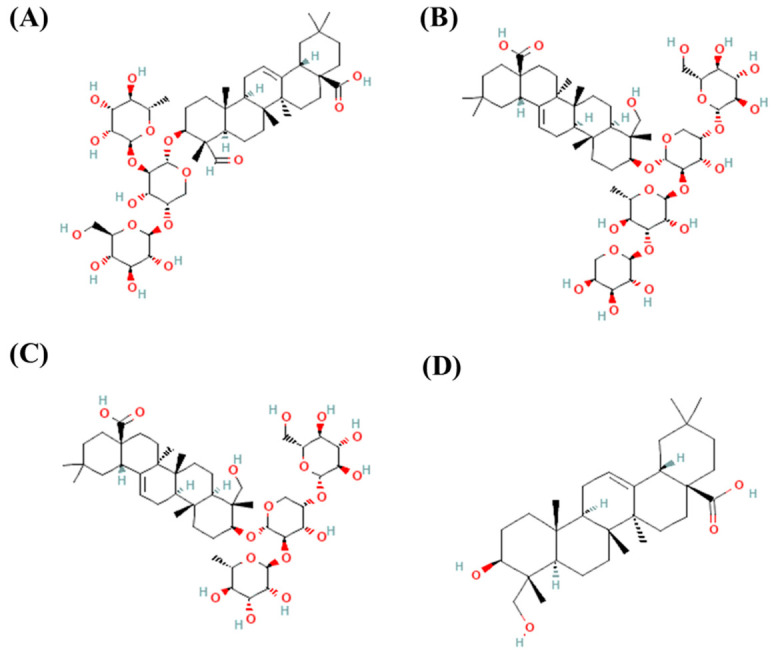
Chemical structures of main saponins reported in *Serjania* species: (**A**) Salzmannianoside A (Compound CID: 21604111), (**B**) Salzmannianoside B (CID: 21604112), (**C**) Pulsatilla saponin D (CID: 11650910), and (**D**) Hederagenin (CID: 73299). The chemical structures and their compound identification (CID) numbers were sourced from PubChem [53].

Saponins are amphiphilic terpenoid secondary metabolites produced by plants, often in response to biotic stress. They consist of a hydrophobic aglycone (sapogenin) linked to one or more hydrophilic sugar moieties. This structural configuration confers surface-active properties and characteristic foaming behavior of saponins. Based on their aglycone structures, saponins are classified as steroidal saponins or terpenoid saponins [57]. These compounds are widely distributed in medicinal plants and are associated with diverse pharmacological activities, including *Serjania* species (Table 2) [58]. The qualitative detection of saponins has been reported in the leaves of *S. marginata* [10,24], *S. erecta* [13,16,29,43], *S. lethalis* [47], and *S. racemosa* [12], as well as in the roots and stems of *S. erecta* [15,16]. These metabolites have been linked to gastroprotective and anti-ulcer effects [10]. In stems of *S. salszmaniana*, several triterpenoid saponins have been isolated, including pulsatilla saponin D, salzmannianoside A, and salzmannianoside B. These compounds demonstrated antifungal and molluscicidal activities [50]. Figure 5 shows the chemical structures of the most representative saponins reported in *Serjania* species. It is important to note that, while most saponins identified in *Serjania* species are found in other plant species [59,60], salzmannianoside A and B have been identified exclusively in *S. salzmaniana*, while 11α-hydroperoxy-hederagenin has been found in *S. triiquetra* [7] and goniocarpic acid in *S. goniocarpa* [21], and, recently, *Serjania racemosa* [12].

### 4.4. Other Compounds

**Figure 6:**
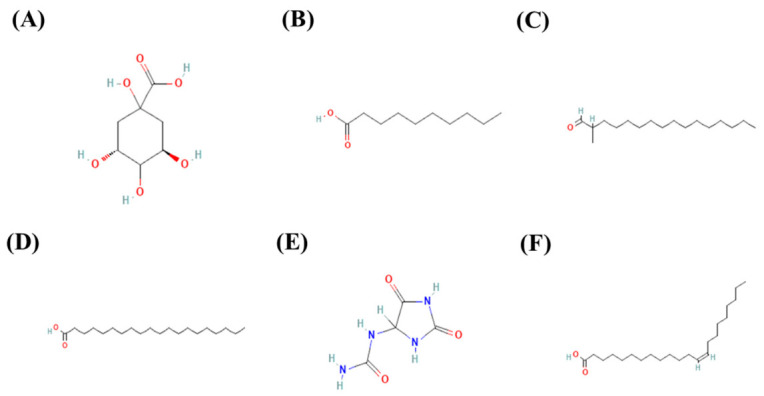
Chemical structures of other compounds reported in *Serjania* species. (**A**) Quinic acid (CID: 6508), (**B**) capric acid (CID: 2969), (**C**) methyl hexadecanal (CID: 546976), (**D**) arachidic acid (CID: 10467), (**E**) allantoin (CID: 204), and (**F**) euricic acid (CID: 5281116). The chemical structures and their compound identification (CID) numbers were sourced from PubChem [53].

Additional bioactive constituents have been reported in several *Serjania* species (Table 2 and Figure 6). Quinic acid has been identified in the leaves of *S. marginata* [44]. In *S. erecta*, cardiac glycosides (roots), steroids (stem and leaves), and fatty acids (seeds), including capric, palmitoleic, oleic, linoleic, α-linoleic, arachidonic, and eicosadienoic acids have been reported [15]. In leaves from *S. lethalis*, hexadecanal, methyl hexadecanoate, hexadecanoic acid, ethyl hexadecanoate, methyl octadecenoate, octadecanoic acid, ethyl octadecenoate, and γ-tocopherol have been identified [30]. Seeds of *S. lethalis* contain palmitic, oleic, linoleic, arachidonic, and eicosanoid acids [48]. Similarly, seeds of *S. salzmaniana* contain palmitic, arachidic, behenic, oleic, eicosanoid, and erucic acids [49]. In *S. caracasana* seeds, palmitic, stearic, arachidic, oleic, eicosanoid, erucic, and linoleic acids have been identified, while allantoin has been reported in leaves [39,49]. In *S. schiedeana* leaves, alkaloids, methyl palmitate, and methyl arachidate have been detected [36,40]. Finally, in stems of *S. triquetra*, ethyl palmitate, stigmasta-3,5-dien-7-one, methyl pentacosanoate, ethyl docosanoate, ethyl oleate, stigmasta-5,22-dien-3-ol, and erucic acid have been reported [6].

## 5. Biological Activities and Biotechnological Applications of *Serjania* Species

In recent years, extracts, fractions, and isolated compounds derived from *Serjania* species have been increasingly investigated for their biological activities and potential biotechnological applications. Although numerous *Serjania* species have been taxonomically documented worldwide, this section focuses exclusively on studies that experimentally evaluated bioactivity or biotechnological utility, including 14 reports for *S. erecta*, 12 reports for *S. marginata*, and 9 reports for *S. lethalis*, as the most extensively investigated species, followed by *S. schiedeana* (4 reports), *S. triquetra* (3 reports), *S. salzmaniana* (2 reports), *S. yucatanensis* (2 reports), *S. caracasana* (2 reports), *S. goniocarpa* (1 report), *S. laruotteana* (1 report), and *S. racemosa* (1 report).

### 5.1. Serjania marginata

Table 3 summarizes the antimicrobial, anti-inflammatory, antinociceptive, antioxidant, cytotoxic, antimutagenic, gastroprotective, insecticidal, and antiparasitic activities reported for extracts derived from leaves and steam of *S. marginata*.

Extracts of *S. marginata* exhibit strain-dependent antimicrobial activity [61,62], as listed in Table 3. Aqueous leaf extracts showed inhibitory effects against *Burkholderia cepacia*, *Enterococcus faecalis*, *Escherichia coli*, *Pseudomonas aeruginosa*, *Staphylococcus epidermidis*, *Staphylococcus aureus*, and *Staphylococcus saprophyticus* in a concentration-dependent response with MIC values ranging from 62.5 to 125 µg/mL; these effects are attributed to the presence of phenols and flavonoids [5]. Hydroalcoholic extracts demonstrated antimicrobial activity against *E. coli*, *S. aureus*, *Salmonella setubal*, *Helicobacter pylori*, and *Candida albicans* with MIC values ranging from 75 to 250 µg/mL [10]. The ethanolic extract showed activity against *Mycobacterium tuberculosis* (MIC: 62.4 µg/mL) but limited or no activity against *Klebsiella pneumoniae*, *S. epidermidis*, and *Pseudomonas aeruginosa* (MIC ≥ 1000 µg/mL) [62]. Additionally, no activity was observed against various *Bacillus* species (*B. toyonensis*, *B. thuringiensis*, *B. cereus*, and *B. proteolyticus*) at concentrations ranging from 1 to 1000 µg/mL [61]. The antimicrobial effects are likely attributed to the synergistic action of phytoconstituents. Polyphenols may interact with membrane proteins or form hydrogen bonds with essential enzymes. Flavonoids can disrupt membrane integrity and interfere with DNA synthesis, and alkaloids may bind microbial DNA, inhibiting transcriptional processes [63,64]. However, some studies did not perform any qualitative or quantitative determinations of bioactive compounds [10,61,62].

The anti-inflammatory effects of *S. marginata* leaf extract have been evaluated using a carrageenan-induced paw edema model in rats (Table 3). Hydroalcoholic extracts (300 mg/kg) reduced edema by up to 58% in a dose-dependent manner [62], while ethanolic extracts (300 mg/kg) achieved approximately 35% inhibition four hours post-administration [11]. The proposed mechanism involves suppression of inflammatory mediators through modulation of the arachidonic acid cascade and NF-κB signaling pathway, possibly mediated by flavonoids (notably rutin), proanthocyanidins, and saponins [11,62], identified via Flow Injection Analysis–Electrospray Ionization–Ion Trap–Mass Spectrometry (FIA-ESI-IT-MS) [11]. The anti-inflammatory effects may also be linked to antioxidant and antihyperalgesic properties, which are associated with the presence of phenols, flavonoids, and tannins [5,11].

The aqueous leaf extract demonstrated significant antinociceptive properties in a formalin-induced nociception murine model (Table 3). A dose of 300 mg/kg reduced nociceptive response by approximately 67%. These effects were attributed to rutin-mediated central modulation involving opioid pathways [11].

Ethanol leaf extracts showed cytotoxic activity against gastric adenocarcinoma cells (100 µg/mL) and non-tumor gastric epithelium cells (300 µg/mL), inducing cell cycle arrest at the G2/M phase [26]. Similar effects were reported in additional gastric cancer models [8,9]. In these studies, no mutagenic effects were detected in normal cells [8,26] or in Salmonella Typhimurium assays with hydroalcoholic extracts [10]. The antimutagenic properties may be linked to the modulation of hepatic xenobiotic-metabolizing enzymes. Additionally, aqueous leaf extracts (224 mg/kg/day) exhibited hepatoprotective effects in *Oreochromis niloticus* (Nile tilapia) [27], further supporting the biological relevance of these extracts (Table 3). Most of these studies did not perform any qualitative or quantitative phytochemical characterization [8,10,26], except for the study conducted by Carmo-Ota et al., who identified quinic acid, quercitrin, isoquercitrin, and proanthocyanidin trimer—A-type, via FIA-ESI-IT-MS [27]. Meanwhile, the extract used by Serpeloni et al. [26] was previously characterized [9]; in total, 15 compounds were identified, including saponins, flavonoids, and proanthocyanidins (Table 2).

Aqueous, ethanolic, and hydroalcoholic leaf extracts exhibited gastroprotective effects in gastric injury models [10,26,27], as listed in Table 3. Hydroalcoholic extracts reduced gastric lesions by 60–90% at 500 mg/kg in murine models [10], associated with decreased myeloperoxidase activity, reduced malondialdehyde levels, and increased mucus production in gastric tissue. Similarly, aqueous extracts (224 mg/kg/day) improved intestinal digestion and reduced gastric damage in Nile tilapia [27]. However, hydroalcoholic extracts (250 mg/kg) did not significantly reduce the severity of diarrhea in a castor oil-induced model [10]. The most beneficial effects were attributed to the phytochemical profile of S. marginata extracts; however, most studies did not perform phytochemical characterization, except for Carmo-Ota et al., who identified several flavonoid compounds [27].

Additionally, leaf and stem extracts demonstrated concentration-dependent photoprotective effects (200–1000 µg/mL) [5]. Both ethanolic and aqueous leaf extracts demonstrated insecticidal activity against *Plutella xylostella*, inhibiting oviposition and increasing larval mortality [32,63]. Moreover, aqueous extract exhibited antiparasitic activity against *Tetrastichus howardi* [28]. Potential bioactivity was attributed to the presence of phytochemicals in *S. marginata* extracts; however, in these studies, the authors did not perform any phytochemical characterization of these extracts [28,32,63], except for Rocha-Falcao et al., who quantified total phenolics and flavonoids by colorimetric chemical reactions [5].

Concerning the toxicity of *S. marginata* extracts, acute oral toxicity studies showed no observable toxicity at 5000 mg/kg in murine models [10]. Subacute administration (2000 mg/kg for 14 days) did not produce overt toxicity; however, renal histological alterations and increased abnormal sperm production were reported [44]. Additionally, aqueous extracts showed no toxicity toward *Artemia salina* at concentrations between 50 and 1000 µg/mL [5].

*Serjania marginata* exhibits diverse biological activities, including antimicrobial, anti-inflammatory, antinociceptive, cytotoxic, and gastroprotective effects, without toxic effects in some models. However, limited phytochemical characterization restricts mechanistic understanding, highlighting the need for compound-level validation.

### 5.2. Serjania erecta

Table 4 summarizes the antimicrobial, antiparasitic, anti-inflammatory, antioxidant, neuroprotective, analgesic, antihypertensive, anti-ulcer, antivenom, insecticidal, and pesticidal activities reported for extracts derived from the leaves, roots, and stems of *S. erecta.*

Extracts of *S. erecta* exhibit concentration- and strain-dependent antimicrobial activity (Table 4). Hydroalcoholic leaf extracts showed inhibitory effects against *Mycoplasma hominis*, *Ureaplasma urealyticum*, and *Mycoplasma arginine* in concentrations ranging from 625 to 2500 µg/mL [65]. Ethanolic extracts from leaves and roots inhibited the growth of *Staphylococcus aureus*, *Pseudomonas aeruginosa*, *Escherichia coli*, *Salmonella setubal*, *Saccharomyces cerevisiae*, and *Candida albicans*; these were strain- and concentration-dependent effects (5–15 µg/mL). However, limited activity was observed against *Mycobacterium tuberculosis* (128 and 256 µg/mL) [17]. The antimicrobial effects were attributed mainly to flavonoids, which are known to disrupt microbial membrane integrity and interfere with nucleic acid synthesis [17]. Additionally, ethanolic leaf extracts demonstrated inhibitory effects against *Bacillus toyonensis*, *B. thuringiensis*, *B. cereus*, and *B. proteolyticus* at concentrations ranging from 1 to 100 µg/mL [58]. Conversely, aqueous leaf extracts did not exhibit antiparasitic activity against *Tetrastichus howardi* [28]. Although the antimicrobial effects were attributed to phytochemicals, most of these studies did not perform phytochemical characterization [28,58,65].

Guimarães et al. reported that the isolated flavonoids (quercetin, vitexin, and isovitexin) from methanolic leaf extracts exerted neuroprotective effects in PC12 cells subjected to Aβ25–35 peptide-induced toxicity. Vitexin exhibited the strongest protective effects (25–200 µg/mL), significantly reducing lactate dehydrogenase release and nitric oxide production (Table 4). These findings suggest that vitexin may attenuate neuronal death associated with β-amyloid–induced oxidative stress, supporting its potential relevance in neurodegenerative disorders [14].

The anti-inflammatory activity of ethanolic leaf extracts was evaluated in a complete Freund’s adjuvant (CFA)-induced paw edema model in rats, where oral administration (100 mg/kg) reduced inflammation by up to 85%; these effects were attributed to the phytochemical profile of *S. erecta* extracts, which were able to decrease the expression of inflammatory proteins via NF-κβ inhibition [4]. Topical hydroalcoholic extracts (0.003–4 mg/ear) demonstrated dose-dependent inhibition of ear edema in murine models [16]. The anti-inflammatory effects were associated with reduced polymorphonuclear leukocyte migration and inhibition of mediators of the arachidonic acid pathway, likely mediated by flavonoids, saponins, tannins, and triterpenoids [13,17,45]. The ethanolic extract also exhibited analgesic activity, which may contribute synergistically to its anti-inflammatory effects [4]. However, no significant anti-inflammatory activity was observed in a rat pulpitis model when an ethanolic leaf extract was evaluated [66], suggesting model-dependent variability.

Maschieto et al. evaluated the effects of a root decoction (5% *w*/*v*, 10 mL/day) in spontaneously hypertensive rats over 32 days. Treatment improved endothelial function and vascular reactivity. The proposed mechanism involves enhanced nitric oxide bioavailability and reduced production of vasoconstrictor factors, potentially mediated by flavonoids, tannins, catechins, and glycosides. Notably, no significant changes in arterial pressure or heart rate were observed [15]. In this study, the phytochemical was qualitatively identified via foam and colorimetric reaction tests.

Methanolic leaf extracts (125–500 mg/kg) significantly reduced ethanol-induced gastric lesions in murine models, with maximal efficacy at 500 mg/kg. The gastroprotective effect appears to involve sensory neurons, nitric oxide pathways, and sulfhydryl group-mediated cytoprotection, reinforcing mucosal defense mechanisms [45]. It must be noted that no phytochemical characterization was performed in this study.

Crude methanolic extracts and fractions from leaves mitigated the toxic effects of *Bothrops jararacussu* venom and its isolated mycotoxins (BthTZ-I and II). The extracts inhibited phospholipase A2 activity, myotoxicity, fibrinogenolysis, hemorrhagic activity, and venom-induced edema. These effects are likely mediated by flavonoids and tannins that chelate metal ions and form complexes with venom proteins, thereby inhibiting metalloproteases, serine proteases, and phospholipases A2 [29].

Additionally, aqueous leaf extracts (5–10% *w*/*v*) exhibited insecticidal activity against *Plutella xylostella*, increasing egg, larval, and pupal mortality. Methanolic extract (0.0078–20 mg/L) demonstrated concentration-dependent pesticidal effects against *Chrysodeixis includens*, extending larval and pupal development [67]. Although these effects were associated with flavonoid and tannin compounds, the authors did not perform any qualitative or quantitative characterizations.

Concerning the toxicity of *S. erecta* aqueous leaf extracts (1250 mg/kg) showed no acute toxicity in a murine model [13]. Similar methanolic and chloroform extracts (5000 mg/kg) did not induce acute oral toxicity in murine models [45]. Root decoction administered for 32 days did not produce macroscopic hepatic alterations in rats [15]. However, aqueous leaf extracts (50 µg/mL) induced morphofunctional and histological alterations in the gills and liver of *Piaractus mesopotamicus*, indicating potential ecotoxicological considerations.

According to these data, *S. erecta* exhibits notable antimicrobial, neuroprotective, and anti-inflammatory properties, primarily attributed to its flavonoid content. Although its gastroprotective and anti-venom effects are promising, the variability in reported efficacy and the absence of systematic phytochemical characterization in the existing literature highlight the need for standardized research.

### 5.3. Serjania lethalis

Extracts and fractions derived from the leaves, roots, and stems of *S. lethalis* have been investigated for their antileishmanial, trypanocidal, larvicidal, antioxidant, and phytotoxic activities, highlighting both pharmacological and agrobiotechnological potential. Alves-Passos et al. reported that the hexane fraction obtained from *S. lethalis* leaves exhibited significant leishmanicidal activity against *Leishmania amazonensis* (IC_50_: 10.29 µg/mL). This fraction was obtained after dispersing the macerated extract (concentrated in a rotatory evaporator at 45 °C) in a methanol: water solution (1:4 *v*/*v*) and subsequent fractionation with hexane. Mechanistically, this fraction induced alterations in parasite cell cycle progression, with a threefold increase in cells in the sub-G0/G1 phase, suggesting apoptosis-like events. Additionally, disruption of mitochondrial membrane potential was observed, indicating mitochondrial dysfunction as a possible mechanism of action [30]. According to the authors, these effects are attributed to the presence of various phytochemicals in hexane fractions, including benzoic acid, α-cubenene, caryophyllene oxide, (E)-phytol, and β-amyrin, among others (Table 2). The root bark extract also demonstrated activity against *Leishmania donovani* promastigotes (IC_50_: 5.2 µg/mL). However, no trypanocidal activity was observed against *Trypanosoma cruzi*, suggesting selective antiparasitic efficacy; although these effects were potentially associated with phytochemicals, the authors did not perform any qualitative or quantitative identification of them [18].

Rodrigues et al. evaluated ethanolic extracts from stem and root bark (plant material previously air-dried, macerated in ethanol at 400 g/L, and concentrated via rotary evaporation under reduced pressure at 40 °C) against *Aedes aegypti* larvae. The root bark extract (resuspended in 1% of dimethyl sulfoxide solution) showed greater larvicidal activity (LC_50_: 285.76 µg/mL) compared to the stem extract (LC_50_: 404 µg/mL). The authors attributed this activity primarily to saponins, which are known to disrupt membrane integrity in insect larvae; however, they did not assess any qualitative or quantitative identification of saponins [68]. Conversely, ethanolic leaf extract (25–150 mg/mL) exhibited limited acaricidal activity against adult female *Dermacentor nitens*, with approximately 30% efficacy [69], indicating modest activity against this equine ectoparasite, which was associated with the presence of flavonoids detected by HPLC-DAD. The extract was obtained by macerating dried leaves (seven days), concentrated by air-forced circulation at 38 °C for 48 h, and resuspended in water prior to evaluation.

Several studies have investigated the phytotoxic potential of *S. lethalis* extracts as an alternative for weed control. Grisi et al. demonstrated that ethanolic crude extract (2.5–10 mg/mL) from the leaves and stems inhibited diaspore germination and seedling growth of wild poinsettia (*Euphorbia heterophylla*) and barnyardgrass (*Echinochloa crus-galli*) in a concentration-dependent manner. Leaf extracts exhibited stronger phytotoxic effects than stem extracts. The inhibition of root elongation in *E. heterophylla* seedlings was attributed to reduced metaxylem cell elongation. Notably, the phytotoxic effects were comparable to or, in some cases, greater than those of commercial herbicides (240 g/L at 1 and 2 L/ha) [70]. This extract was obtained by macerating dried leaves in ethanol (100 g/500 mL for 72 h) and concentrated in a rotatory evaporator under reduced pressure. Then, the extract was resuspended in buffer solution and DMSO. Similarly, crude ethanolic leaf extracts (obtained via maceration at 100 g/L and concentrated in a rotatory evaporator under reduced pressure) and derived fractions (resuspended in 5% DMSO solution at 0.8 mg/mL) have been reported to inhibit metaxylem elongation in *Sesamum indicum* roots [38]. It must be noted that, in these studies, the authors did not undertake any qualitative or quantitative identification of bioactive molecules [38,70].

Concentrated aqueous leaf extracts (2.5–10%) reduced the germination and seedling growth of *Panucum maximum* in a concentration-dependent manner [71]. Additionally, aqueous extracts from leaves and stems inhibited germination and early growth of *Sesamum indicum*, *Raphanus sativus*, and *Triticum aestivum* [19]. Saponin-rich fractions (0.2–0.8 mg/mL) obtained from leaves, roots, and stems demonstrated inhibitory effects on coleoptile elongation, with root and leaf fractions exhibiting the strongest activity [72]. Collectively, these findings suggest that *S. lethalis* extracts represent a promising alternative for developing plant-derived bioherbicides and sustainable weed management strategies. In these studies, extracts were obtained via the maceration (100 g of dried leaves in 1 L of water at 4 °C for 24 h) and then concentrated under vacuum in a rotary evaporator [19,71,72]. Nonetheless, a saponin-rich fraction was obtained by fractionating the aqueous leaf extract with methanol, which was dried and resuspended with water [72]. Although the effects, direct or indirect, were associated with the presence of phytochemicals, it must be noted that, in these studies, the authors did not assess any qualitative or quantitative identification of phytochemicals [19,71,72].

Cavalcante et al. reported that seed oil obtained via hydrodistillation from *S. lethalis* exhibited antioxidant activity, as evidenced by free radical scavenging assays against DPPH (EC_50_: 17.6 µg/mL) and ABTS radical cation (EC50: 22.5 µg/mL) [20]. These results support the contribution of lipid-soluble phytoconstituents (α-thujene, δ-terpinene, methyl ether thymol, thymol, carvacrol, and β-caryophyllene) to the species’ antioxidant profile.

Other *Serjania* species are predominantly investigated for their potential applications and biological activities. In this context, studies on *S. lethalis* extracts and fractions are focused on evaluating antiparasitic, larvicidal, phytotoxic, and antioxidant activities, positioning it as a promising source of agrobiotechnological products. However, the limited phytochemical characterization across studies highlights a critical gap that must be addressed to fully elucidate its mechanisms of action and enable its rational application.

### 5.4. Serjania salszmaniana

Ekabo and Farnsworth isolated two saponins from the methanolic stem extract of *S. salzmanniana* and evaluated their antifungal and molluscicidal activities. The isolated compounds (identified via nuclear magnetic resonance) exhibited significant antifungal activity against *Cryptococcus neoformans* (MIC: 8 µg/mL) and *Candida albicans* (MIC: 16 µg/mL), while showing no activity against *Aspergillus fumigatus* (MIC > 250 µg/mL). In addition, the identified saponins (salzmannianoside A and B, pulsatiila saponin D, and 3-*O*-[[β-D-Glucopyranosyl-(1→4)-[r-α-rhamnopyranosyl-(1→2)]-α-L-arabinopyrnoyl] oleanolic acid) displayed molluscicidal activity against *Biomphalaria alexandrina* at 100 ppm, inducing 70–100% mortality after 48 h of exposure [50].

Additionally, the fatty acid profile of *S. salzmanniana* seed oil has been reported to be suitable for biodiesel production, highlighting its potential as a renewable bioenergy source [49].

According to these data, *S. salzmanniana* exhibits notable antifungal and molluscicidal activities, which can be attributed to saponins, and it has potential as a renewable bioenergy source due to its seed oil profile. However, the scarce number of studies available underscores the need for further research to validate and expand its biotechnological applications.

### 5.5. Serjania yucatanensis

Chagas disease, caused by the flagellate protozoan *Trypanosoma cruzi*, remains a major public health concern in Latin America and is increasingly a concern in non-endemic regions due to migration. Although antiparasitic drugs such as benznidazole and nifurtimox are available, their efficacy is limited during the chronic phase and may be associated with adverse effects. The ethanolic leaf extract of *S. yucatanensis* was obtained via maceration (20 g of dried leaves in 400 mL of ethanol for 72 h) and concentrated under reduced pressure. The extract was resuspended in DMSO prior to analysis. This extract has demonstrated in vitro trypanocidal activity against epimastigote and trypomastigote forms of two *T. cruzi* strains (Y and Ninoa), reducing parasite numbers in a concentration-dependent manner (50–100 µg/mL). In a murine model, oral administration of the extract (100 mg/kg) achieved a 75% reduction in parasitemia without evident toxicity, surpassing the efficacy of allopurinol, the positive control. In vitro assays conducted on infected Vero cells showed that the extract interfered with parasite egress and reduced the number of trypomastigotes, suggesting disruption of critical steps in the parasite life cycle. However, further studies are required to assess efficacy during the chronic phase of the infection, where current therapies are less effective [73]. Subsequently, the same research group compared the activity of the crude ethanolic extract (IC_50_: 38.2 µg/mL), the hexane fraction (IC_50_: 78 µg/mL), and a fraction enriched in lup-20(29)-en-3-one and β-caryophyllene oxide (IC_50_: 80.3 µg/mL). The crude extract demonstrated superior trypanocidal activity, suggesting possible synergistic interactions among phytoconstituents. Importantly, no cytotoxic effects were observed in Vero cells at 100 µg/mL [22]. The crude ethanolic extract was obtained by maceration (428 g of dried leaves in 6 L of ethanol for 72 h) and concentrated under reduced pressure, prior to fractionation with hexane and ethyl acetate, which were then purified via vacuum liquid chromatography.

Additionally, the fatty acid profile of *S. caracasana* seed oil has been reported to be suitable for biodiesel production, highlighting its potential as a renewable bioenergy source [49].

In summary, *S. yucatanensis* exhibits promising trypanocidal activity both in vitro and in vivo, likely driven by synergistic interactions among its phytoconstituents, with no evident cytotoxicity. However, the limited number of studies and lack of evaluation in the chronic phase of infection highlight the need for further investigation to confirm its therapeutic potential.

### 5.6. Serjania caracasana

In traditional medicinal practice, decoctions prepared from the leaves of *S. caracasana* are commonly used to treat gastric disorders [74]. In this context, both gastroprotective and antispasmodic activities have been experimentally investigated [39,74]. The ethanolic leaf extract (obtained by maceration of dried aerial parts and concentrated by evaporation) demonstrated gastroprotective effects in a murine model of ethanol-induced gastric ulcer, showing a dose-dependent response (50, 150, and 500 mg/kg) comparable to that of ranitidine. Additionally, the extract exhibited in vitro antispasmodic activity in rat ileal preparations pre-contracted with KCl, suggesting calcium-channel-mediated smooth muscle relaxation [74]. In this study, the dried extract was dissolved in 3% cremophor and diluted in MiliQ water (10 mg/mL) for in vitro experiments, whereas it was dissolved in Twen-20 (0.32 mg/mL) and diluted with distilled water for in vivo studies. Similarly, Silva et al. reported that the hexane (IC_50_: 68.4 µg/mL), dichloromethane (IC_50_: 161.34 µg/mL), and butanol (IC_50_: 219.8 µg/mL) fractions obtained from aerial parts of *S. caracasana* exerted antispasmodic effects in rat ileum assays. The butanol fraction exhibited low hemolytic activity (≤2%), indicating a favorable preliminary profile. Furthermore, the crude ethanolic extract was non-toxic in murine models following oral administration at 2000 mg/kg. The observed biological activities have been attributed to phytoconstituents such as β-amyrin, allantoin, quercitrin, and friedelin, which were identified via NMR and GC-MS spectroscopy [39]. For fractionation and compound isolation, first, a maceration of air-dried aerial parts (1916 g of dried material in 96% ethanol at room temperature) was performed, and the resulting solution was concentrated under reduced pressure at 40 °C. Then, the ethanolic extract (200 g) was resuspended in a 70:30 (*v*/*v*) methanol–water solution and subsequently fractionated with butanol, hexane, and dichloromethane after drying.

Additionally, the fatty acid profile of *S. caracasana* seed oil (highlighting eicosenoic acid) has been reported to be suitable for biodiesel production and could serve as a bioenergetic source [49].

In summary, these findings highlight *S. caracasana* as a promising source of gastroprotective and antispasmodic agents, consistent with its ethnobotanical use, and they also indicate biotechnological potential for bioenergy applications through its seed oil. Nevertheless, comprehensive studies focusing on phytochemical characterization, mechanisms of action, and pharmacokinetics are still required to fully validate its therapeutic use.

### 5.7. Serjania goniocarpa

Quintal-Novelo et al. isolated goniocarpic acid from the leaves of *S. goniocarpa* from hexane fractions (obtained from previous maceration of 3 kg of dried leaves in 15 L of methanol for 72 h at room temperature and concentrated under reduced pressure) and evaluated its cytotoxic and antiproliferative effects against several human cancer cell lines. The compound exhibited activity against HeLa (IC_50_: 2.0 and 16.1 µg/mL), Hep-2 (IC_50_: 5.3 and 8.7 µg/mL), MCF-7 (IC_50_: 2.5 and 7.8 µg/mL), KB (IC50: 1.4 and 3.4 µg/mL), PC3 (IC50: 11.3 and 45.5 µg/mL), and Hek-293 (IC50: 9.3 and 15.3 µg/mL), with responses varying according to the cell line. Although these IC_50_ values were higher than those reported for docetaxel (IC_50_: 0.01–0.2 and 0.02–0.05 µg/mL, respectively), the results indicate moderate cytotoxic potency. The authors proposed that goniocarpic acid may serve as a scaffold for structural optimization rather than as a direct chemotherapeutic candidate [21].

Although goniocarpic acid, isolated from *S. goniocarpa*, exhibits moderate cytotoxic and antiproliferative activities, the current evidence is limited to a single study, underscoring the need for further research to validate and expand its therapeutic potential.

### 5.8. Serjania laruotteana

Silva-Ribeiro et al. studied the endophytic fungal community associated with *S. laruotteana*. A total of 261 fungal isolates were obtained, of which 58 strains were taxonomically identified. The most prevalent genera included *Colletotrichum* and *Diaporthe*, while additional isolates belonged to *Xylaria*, *Phyllosticta*, *Muyocopron*, *Fusarium*, *Nemania*, *Plectosphaerella*, *Corynespora*, *Bipolaris*, and *Curvularia*. Endophytic fungi are recognized as important sources of diverse bioactive metabolites. Therefore, the microbial community associated with *S. laruotteana* may represent an indirect but valuable reservoir of compounds with pharmaceutical and biotechnological applications [33]. However, the evidence is currently limited to a single study, emphasizing the need for further research to explore and validate this microbial reservoir.

### 5.9. Serjania schiedeana

Extracts of *S. schiedeana* have been evaluated for anti-inflammatory and insecticidal activities, supporting both pharmacological and agrobiotechnological applications [36,37,40,51].

Salinas-Sánchez et al. assessed the anti-inflammatory activity of ethyl acetate extracts and an ethyl acetate fraction derived from *S. schiedeana* using a TPA-induced ear edema murine model and a kaolin/carrageenan (K/C)-induced arthritis murine model. In the TPA-induced model, the crude extract produced 90% inhibition of auricular edema at 2 mg/ear, while the fractions achieved inhibition rates between 67% and 89%. A proanthocyanidin-type compound, identified as epicatechin–(4β → 8)–epicatechin–(4β → 8, 2β → O → 7) epicatechin, was isolated and showed 72% inhibition of edema (ED_50_: 0.25 mg/ear, Emax: 52.9%). In the K/C-induced arthritis model, the ethyl acetate extract (400 mg/kg) and the proanthocyanidin-rich fraction (10 mg/kg) significantly reduced inflammation from the first day of administration, achieving reductions of 94% and 62%, respectively. Treatment with *S. schiedeana* extracts decreased levels of interleukin (IL)-1β, IL-17, and IL-6. At a higher dose, the ethyl acetate extract also reduced tumor necrosis factor-α (TNF-α) and IL-10 concentrations. The anti-inflammatory effects are likely associated with the inhibition of arachidonic acid metabolism via cyclooxygenase (COX) and lipoxygenase (LOX) pathways, as well as the modulation of pro-inflammatory cytokines. These findings support the traditional use of *S. schiedeana* in the treatment of inflammatory conditions [40].

The insecticidal activity of aqueous and ethyl acetate fractions from *S. schiedeana* stems has been evaluated against L1 larvae of *Spodoptera frugiperda* and adult apterous *Melanaphis sacchari* [36,37,51]. The aqueous fraction (obtained from previous maceration of 500 g of dried material in 2.5 L of methanol for three days and concentrated to dryness in a rotatory evaporator prior to fractionating) demonstrated aphidicidal activity against adult female *M. sacchari* in a concentration-dependent manner (1000–10,000 ppm), producing mortality rates between 34% and 82% (LC_50_: 3013 ppm) after 72 h [36]. Similarly, the ethyl acetate fraction induced 72% mortality in adult apterous *M. sacchari* at 10,000 ppm after 72 h [51]. In addition, aqueous stem extracts (250 and 1000 ppm) exhibited insecticidal activities against L1 larvae of *S. frugiperda* under laboratory and greenhouse conditions. The extract reduced larval weight by up to 50%, increased mortality above 50%, and resulted in complete larval and pupal mortality at higher concentrations. Foliar damage in maize plants was reduced by up to 18% after seven days of exposure [37]. The insecticidal activity of aqueous fraction and aqueous extract was attributed to alkaloids, flavonoids, and tannins, which were detected via colorimetric reactions [36,37], while the effects of ethyl acetate fraction were associated with the presence of methyl palmitate and other compounds with low polarity [51]. These findings highlight the potential of *S. schiedeana* as a source for developing bioinsecticides as an alternative to synthetic pesticides.

In summary, *S. schiedeana* exhibits potent anti-inflammatory and notable insecticidal activities, supporting its dual pharmacological and agrobiotechnological potential. However, the limited number of studies highlights the need for further research to confirm its efficacy, deepen phytochemical characterization, and elucidate its mechanisms of action.

### 5.10. Serjania triquetra

In traditional medicine, decoctions from leaves and stems of *S. triquetra* are used to treat microbial infections. Navarro et al. evaluated the antimicrobial efficacy of methanolic stem extracts against *S. aureus*, *E. coli*, *P. aeruginosa*, and *C. albicans*. Their findings showed that *S. aureus* exhibited the highest sensitivity (MIC: 20 mg/mL), whereas the remaining microorganisms showed limited susceptibility (MIC > 40 mg/mL). These relatively high MIC values suggest moderate antimicrobial potency, warranting further phytochemical characterization and mechanistic studies [23]. The extract was obtained via maceration of 100 g of dried material in 1500 mL of methanol for five days and then concentrated under reduced pressure in a rotatory evaporator until dryness and resuspended in 10% Tween 80 prior to in vitro evaluation.

The vasorelaxant activity of concentrated hydroalcoholic stem extracts (0.0011–100 µg/mL) obtained via maceration was evaluated in isolated rat aortic rings. The effect was concentration dependent and endothelium dependent, suggesting involvement of nitric-oxide-mediated pathways. The extract reduced peripheral vascular resistance and produced hypotensive effects comparable to, lower than, or, in some cases, greater than those of carbachol and nifedipine [6]. The vascular activity has been tentatively attributed to ursolic acid and allantoin, which were identified via chromatography and NMR analysis, although definitive mechanistic confirmation remains necessary.

Regarding toxicity, hydroalcoholic extracts from commercial preparations of *S. triquetra* showed no toxicity in *Artemia salina* assays (LD_50_ ≥ 1000 µg/mL), suggesting low acute toxicity under the tested conditions [75].

According to these data, *S. triquetra* exhibits moderate antimicrobial activity, along with notable vasorelaxant and hypotensive effects, and low acute toxicity, supporting its ethnobotanical use. However, the limited number of studies highlights the need for further research to elucidate its phytochemical profile and underlying mechanisms of action.

### 5.11. Serjania racemosa

Research on *S. racemosa* extracts has systematically assessed their antioxidant, antimicrobial, and antiproliferative effects [12]. In this specific *Serjania* species, NMR spectroscopy has identified saponins, flavonoids, and glycosylated flavonoids in methanol, ethyl acetate, and hexane extracts. These extracts have shown the ability to inhibit free radicals by 22% to 91%, as determined by the DPPH test, and to reduce ferric ions, with FRAP test results ranging from 54 to 520 µmol Fe^+2^, depending on the extraction solvent used (methanol > ethyl acetate > hexane). In terms of antimicrobial activity, the methanolic extract demonstrated the strongest inhibitory effect (10 mg/mL) against *E. coli*, *Salmonella* Typhi, and *K. pneumoniae*, while the ethyl acetate and hexane extracts were effective against *Proteus mirabilis*. These findings were compared to ceftriaxone. However, the extracts did not show activity against *Burkholderia cepacia*, *Candida albicans*, *Candida tropicalis*, and *Candida krusei*. The antiproliferative activity was tested on the androgen-independent prostate cancer cell line (PC-3), showing a concentration-dependent reduction in cell proliferation, influenced by the type of extract used. According to the authors, at concentrations of 700 and 1000 µg/mL, the ethyl acetate extract significantly impacts the proliferation of the PC-3 prostate cancer cell line, likely through a cytotoxic mechanism.

Further research is needed to identify the secondary metabolites responsible for these effects and to assess the toxicity of the extracts to ensure their safety and effectiveness.

## 6. Challenges and Prospects of *Serjania* Species

Despite the growing body of research on *Serjania* species, the current state of knowledge still presents significant limitations that hinder the effective translation of their findings into pharmacological and biotechnological applications. These limitations arise largely from the predominance of studies using crude extracts, in which biological activities are reported without comprehensive identification or quantification of the compounds responsible. Although associations between phytochemical composition and biological effects can be suggested, such relationships remain largely tentative and should be interpreted with caution.

Another important limitation is the notable gap between the genus’s extensive botanical diversity and the relatively small number of species that have been investigated from phytochemical, pharmacological, or biotechnological perspectives. Numerous species, including *Serjania* species such as *S. communis* [76], *S. lucianoi* [77], *S. recemosa* [78], *S. adenphylla* [79], *S. comata* [80], *S. corrugata* [81], *S. fuscifolia* [82], *S. littoralis* [83], *S. meridionalis* [84], *S. mexicana* [85], *S. pygmaea* [86], *S. rzedowskiana* [87], *S. setlgera* [88], *S. rosalindae*, and *S. crucensis* [89], have been taxonomically described but remain largely unexplored in terms of chemical composition and biological potential.

From a pharmaceutical perspective, most reported bioactivities, particularly for *S. marginata*, *S. erecta*, and *S. lethalis*, are based on crude extracts rather than isolated compounds. Therefore, there is a clear need to advance toward bioassay-guided fractionation strategies that enable the isolation and identification of the molecules responsible for these effects. In parallel, further investigations at the molecular level are required, including the identification of biological targets, modulation of signaling pathways, and the establishment of structure–activity relationships.

Although several *Serjania* species have demonstrated low acute toxicity, reports of subacute effects, such as alterations in renal histology and reproductive parameters, highlight the need for comprehensive toxicological evaluation, including chronic exposure studies, genotoxicity, and pharmacokinetic profiling. Rigorous safety assessment is essential for the potential translation of these botanical preparations into evidence-based therapeutic agents. For example, in Mexico, some *Serjania* species are marketed online as herbal remedies in tea bags, with their recommended uses grounded in traditional practices and ancestral knowledge. However, these products are primarily supported by traditional knowledge rather than robust clinical validation. The absence of standardized extraction protocols, quality control measures, and clinical trials underscores the need for regulatory and scientific frameworks to ensure safety, efficacy, and reproducibility.

From a biotechnological perspective, *Serjania* species could be a source of bioherbicides and biopesticides for sustainable crop management. Additionally, the fatty acid profile of seeds from some species highlights their potential for biodiesel production. However, their practical application remains limited by the lack of standardized formulations and validation, highlighting the need for translational research. Additionally, the endophytic microorganisms associated with certain species may serve as alternative sources of diverse bioactive compounds.

Bridging this gap will require a shift from predominantly descriptive studies toward integrative and mechanism-oriented research frameworks. The implementation of such approaches will be critical not only for elucidating the functional relevance of *Serjania* phytochemistry but also for unlocking its full potential in pharmacological and agrobiotechnological applications.

## 7. Conclusions

Although the phytochemical diversity and biological activities of *Serjania* species have been relatively underexplored, they exhibit considerable potential as a valuable source of bioactive compounds with pharmacological and biotechnological applications. In particular, this relates to some saponin compounds that are found exclusively in *Serjania* species.

Evidence indicates that several *Serjania* species, particularly those more extensively studied, exhibit promising antioxidant, anti-inflammatory, antimicrobial, and cytotoxic properties, largely attributable to the presence of saponins, flavonoids, and other secondary metabolites. Despite these advances, the current body of literature remains unevenly distributed across species and research areas, with a predominance of in vitro studies and limited in vivo and clinical validation. Therefore, further in-depth investigations and translational approaches are required to fully elucidate their therapeutic potential and facilitate their integration into pharmaceutical and biotechnological applications.

## Figures and Tables

**Table 1:** Claimed ethnobotanical uses for different plant parts of the *Serjania* species.

*Serjania* Species	Common Name	Country or Region	Claimed Therapeutic Use	Plant Part	Preparation/Application	Ref.
*S. marginata*	Cipó-uva	Brazil Argentina Paraguay Bolivia	Gastrointestinal disorders, ulcers, cancer, and infections	Leaves	Decoction/Oral	[8,9,10,11]
*S. erecta*	Cipó-cinco-folhas, cinco-folhas	Brazil	Inflammation, stomachache, ulcerative diseases, hypertension, gastritis, pain management, and back pain	Stems Leaves Roots	Decoction/Oral	[4,13,14,15,16,17,29]
*S. lethalis*	Timbo vine cipó-timbó timbó	Brazil	Inflammation, infections, skin diseases, ulcers, diarrhea, fever, malaria, kidney pain, narcotic	Stems Leaves	Decoction/Oral	[18,19,20,38]
*S. caracasana*	Tingui-da-mata	Brazil	Gastric problems	No information	No information	[39]
*S. goniocarpa*	*But aak*	Mexico	Leg sores, abscesses	No information	No information	[21]
*S. schiedeana*	Costilla de vieja, Cuapalachtle	Mexico	Kidney inflammation, burning feet, back pain, healing wounds, and bruises	No information	No information	[37,40]
*S. yucatenensis*	*Chéen peek* *Chac uayam*	Mexico	Abscesses, infections, vomiting, headache, diarrhea	Stems Leaves	Decoction/Oral	[22]
*S. triquetra*	Palo de tres costillas, bejuco de tres costillas, tres equis	Mexico	Kidney pain, kidney stones, diuretic agents, hepatitis, urinary infections, and kidney inflammation	Stems Leaves	Decoction/Oral	[6,7,23]
*S. racemosa*	Seven-hearted bejuco	Mexico	Treatment of diabetes, diuretic, kidney problems, kidney inflammation, urinary problems, and prostate disorders	No information	No information	[12]

**Table 2:** Bioactive compounds content in leaves, roots, and stems of different *Serjania* species.

*Serjania* Species	Plant Part	Extraction Method	Solvent/Liquid-to-Solid Ratio	Detection/Quantification Method	Reported Compound	Classification	Identified/ Content	Ref.
*S. marginata*	Leaves	Percolation	Ethanol, then the extract was fractionated	NMR	3-O-D-β-glucopyranosylsitosterol	Phytosterol glycoside	Identified	[9]
					Pulsatilla saponin D	Saponin	Identified	
					Hederacolchiside A	Saponin	Identified	
					Salzmannianoside B	Saponin	Identified	
					Quercetin 3-O-α-L-rhamnopyranoside	Flavonoid glycoside	Identified	
					Epicatechin	Flavanol	Identified	
					Cassiaoccidentalin A	Flavonoid glycoside	Identified	
					Tetrastigma B	Flavonoid glycoside	Identified	
					Apigenin 6-C-β- boivinopyranosyl-7-O-β-D-glucopyranoside	Flavone glycoside	Identified	
					apigenin 6-C-[2- O-α-L-rhamnopyranosyl(1→2)]-β-D-xylopyranoside	Flavone glycoside	Identified	
					proanthocyanidins A-1	Anthocyanin	Identified	
					proanthocyanidins A-2	Anthocyanin	Identified	
					Cinnamtannin B-1	Protoanthocyanidin	Identified	
	Leaves	Percolation	70% ethanol solution	TLC HPLC	Saponins	Terpene	Identified	[10]
					Flavonoid glycosides	Phenolic compound	Identified	
					Tannins	Polyphenol	Identified	
	Leaves	Maceration	Distilled water (10:1 mL/g)	FIA-ESI-IT-MS	Soluble phenols	Phenolic compound	Identified	[11]
					Flavonoids	Phenolic compound	Identified	
					Tannins	Polyphenol	Identified	
					Rutin	Flavonol glycoside	Identified	
	Leaves	Ultrasound	70% ethanol solution (10:1000 mL/mg)	HPLC-PDA	Protocatechuic acid	Phenolic acid	Identified	[25]
					(epi)catechin-(epi)catechin	Flavanol	Identified	
					(epi)catechin-A-(epi)catechin-(epi)catechin	Flavanol	Identified	
					(epi)catechin	Flavanol	Identified	
					(epi)catechin-(epi)catechin-(epi)catechin	Flavanol	Identified	
					(epi)catechin-A-(epi)catechin	Flavanol	Identified	
					Quercetin-*O*- hexoside	Flavonol glycoside	Identified	
					Apigenin-*C*-[2″-*O*-(deoxyhexosyl)- pentoside]	Flavone glycoside	Identified	
					Quercetin-*O*-deoxyhexoside	Flavonol glycoside	Identified	
					Luteolin-6-*C*-[2″-*O*-(deoxyhexosyl)-hexos-3-uloside] (cassiaoccidentalin B)	Flavone glycoside	Identified	
					Luteolin-8-*C*-[2″-*O*-(deoxyhexosyl)-hexos-3-uloside]	Flavone glycoside	Identified	
					Apigenin-6-*C*-[2″-*O*-(deoxyhexosyl)-hexos-3-uloside] (cassiaoccidentalin A)	Flavone glycoside	Identified	
					Apigenin-8-*C*-[2″-*O*-(deoxyhexosyl)-hexos-3-uloside] (tetrastigma B)	Flavone glycoside	Identified	
					Apigenin-*C*-hexos-3-uloside-2″-*O*-deoxyhexosyl	Flavone glycoside	Identified	
					*O*-deoxyhexosyl-*C*-glycosyde apigenin derivative	Flavone glycoside	Identified	
					Methyl-luteolin-6-*C*-[2″-*O*-(deoxyhexosyl)-hexos-3-uloside] (cassiaoccidentalin C)	Flavone glycoside	Identified	
					*O*-deoxyhexosyl apigenin derivative	Flavone glycoside	Identified	
					Methyl-luteolin-8-*C*-[2″-*O*-(deoxyhexosyl)-hexos-3-uloside]	Flavone glycoside	Identified	
	Leaves	Maceration	Water (20 mL/2 g)	FIA-ESI-IT-MS	Quinic acid	Organic acid	Identified	[27]
					Quercitrin	Flavonol glycoside	Identified	
					Isoquercitrin	Flavonol glycoside	Identified	
					Proanthocyanidin trimer—A-type	Tannin	Identified	
	Leaves	Maceration	Water: (50:2 mL/g)	Mass spectrometry	Soluble phenols	Phenolic compound	266.70 mg/g DM	[44]
					Flavonoids	Phenolic compound	189.10 mg/g DM	
					Tannins	Phenolic compound	56.3 mg/g DM	
					Quinic acid	Carboxylic acid	Identified	
					Quercetin-3- *O*-rhamnoside (quercitrin	Flavonol glycoside	Identified	
					Quercetin-3-*O*-glucose (isoquercetin)	Flavonol glycoside	Identified	
					Proanthocyanidin trimer-A.type	Anthocyanin	Identified	
	Leaves	Ultrasound	70% ethanol solution (1:100 mL/mg)	UHPLC-(ESI)-HRMS NMR	Phenolic acids	Phenolic compound	Identified	[24]
					Cinnamic acids	Phenolic acid	Identified	
					Triterpene	Terpene	Identified	
					B-type proanthoccyanidins	Anthocyanin	Identified	
					B-type proanthoccyanidins trimer	Anthocyanin	Identified	
					B-type proanthoccyanidins tretamer	Anthocyanin	Identified	
					A-type proanthoccyanidins dimer	Anthocyanin	Identified	
					A-type proanthoccyanidins trimer	Anthocyanin	Identified	
					A-type proanthoccyanidins tretamer	Anthocyanin	Identified	
					A-type proanthoccyanidins pentamer	Anthocyanin	Identified	
					Flavonoids glycosylated	Phenolic compound	Identified	
					*C*-glycosylated flavone	Flavone glycoside	Identified	
					*C*,*O*-glycosylated flavone	Flavone glycoside	Identified	
					*O*-glycosylated flavone	Flavone glycoside	Identified	
	Leaves and stems	Maceration	Distilled water (200:20 mL/g)	Spectrometric techniques	Phenolic compounds	Phenolic compound	195–300 mg/g DM	[5]
					Flavonoids	Phenolic compound	7–255 mg/g DM	
					Tannins	Polyphenol	248–265 mg/g DM	
*S.erecta*	Roots	Decoction	Water	Colorimetric test	Flavonoids	Phenolic compound	Identified	[15]
					Saponins	Terpene	Identified	
					Tannis	Polyphenol	Identified	
					Catechins	Flavanol	Identified	
					Cardiac glycosides	Glycoside compound	Identified	
	Steams and leaves	Maceration	Methanol	Column chromatographic	Saponins	Terpene	Identified	[29]
					Terpenes	---	Identified	
					Flavonoids	Polyphenol	Identified	
					Tannins	Polyphenol	Identified	
	Leaves			Foam test	Saponins	Terpene	Identified	[43]
				Spectrophotometric techniques	Flavonoids	Flavonoids	212 mg/g DM	
					Phenolic compounds	Phenolic compounds	386 mg/g DM	
					Tannins	Tannins	89 mg/g DM	
	Leaves				Polyisoprenoids	Terpene	Identified	[45]
	Leaves	Sequential extraction	N- hexane, ethyl acetate, ethanol	NMR FTIR TLC	(−)-epicatechin	Flavanol	Identified	[4]
					kaempferol	Flavonol	Identified	
					Isovitexin	Flavone glycoside	Identified	
					apigenin-8-C-β-D-glucopyranoside	Flavone glycoside	Identified	
					kaempferol-3,7-di-O-α-L-rhamnopyranoside	Flavonol glycoside	Identified	
					kaempferol-3-*O*-α-L-rhamnopyranoside	Flavonol glycoside	Identified	
	Leaves	Maceration	Ethanol	NMR	Kaempferol	Flavonol	Identified	[17]
					Kaempferol-3,7-di-*O*-a-L-rhamnopyranoside	Flavonol glycoside	Identified	
					(-)-epicatechin	Flavanol	Identified	
					Apigenin-6-C-b-D-glucopyranoside (isovitexin)	Flavone glycoside	Identified	
					apigenin-8-C-b-D-glucopyranoside (vitexin)	Flavone glycoside	Identified	
					kaempferol-3-*O*-a-l-rhamnopyranoside	Flavonol glycoside	Identified	
					kaempferol-3-*O*-a-l-rhamnopyranosyl-(1→6)-b-d- glucopyranoside	Flavonol glycoside	Identified	
	Leaves	Decoction	Distilled boiling water (90 g/900 mL)	TLC HPLC	Saponins	Terpene	Identified	[13]
					Tannins	Polyphenol	Identified	
					Glycosidic flavonoids	Phenolic compound	Identified	
	Leaves/stems	Maceration	70% ethanol solution	No information	Saponins	Terpene	Identified	[16]
					Flavonoids	Polyphenol	Identified	
					Triterpenoids	Terpenes	Identified	
					Steroids	Terpene	Identified	
					Tannins	Polyphenol	Identified	
					Catechins	Flavanol	Identified	
	Shrub	Homogenization	2:1 *v*/*v* Chloroform:methanol solution	Gas chromatography	Capric acid	Fatty acid	3.0 g/100 g DM	[46]
					Palmitoleic acid	Fatty acid	0.19 g/100 g DM	
					Oleic acid	Fatty acid	1.33 g/100 g DM	
					Linoleic acid	Fatty acid	0.20 g/100 g DM	
					α-Linoleic acid	Fatty acid	0.11 g/100 g DM	
					Arachidonic acid	Fatty acid	0.98 g/100g DM	
					Eicosadienoic acid	Fatty acid	6.23 g/100 g DM	
	Leaves	Maceration	Methanol	TLC HPLC	Isovitexin	Flavone glycoside	Identified	[14]
					Vitexin	Flavone glycoside	Identified	
					Quercetin	Flavonol	Identified	
*S. lethalis*	Leaves	Static extraction	Distilled water	HPLC	Flavonoids	Polyphenol	Identified	[47]
					Saponins	Terpene	Identified	
					Terpenoids	Terpene	Identified	
	Leaves	Maceration	90:10 *v*/*v* ethanol-water	GC-MS GC-FID	Benzoic acid	Phenolic acid	Identified	[30]
					α-Cubene	Monoterpene	Identified	
					4-epi-cubedol	Monoterpene	Identified	
					(-)-Spathulenol	Monoterpene	Identified	
					Caryophyllene oxide	Sesquiterpene	Identified	
					Conifery alcohol	Monolignol	Identified	
					Hexadecanal	Fatty aldehyde	Identified	
					6,10,14-Trymethyl-2-pentadecacone	Ketone	Identified	
					Phytol	Diterpene alcohol	Identified	
					Methyl hexadecanoate	Fatty acid ester	Identified	
					Hexadecanoic acid	Fatty acid	Identified	
					Ethyl hexadecanoate	Fatty acid ester	Identified	
					(E)-Phytol	Diterpene	Identified	
					Methyl octadecanoate	Fatty acid ester	Identified	
					Octadecanoic acid	Fatty acid	Identified	
					Phytol acetate	Diterpenes	Identified	
					Ethyl octadecenoate	Fatty acid ester	Identified	
					4,8,12,16-Tretamethylheptadecan-4-olide	Fatty alcohol	Identified	
					γ-Tocopherol	Monoterpene	Identified	
					β-Amyrone	Triterpene	Identified	
					β-Amyrin	Triterpene	Identified	
					Lup-20(29)-en-3-one	Triterpene	Identified	
					Lup-20(29)-en-3-ol	Triterpene	Identified	
					Glutinone	Triterpene	Identified	
					β-Amyrin acetate	Triterpene	Identified	
	Leaves	Hydrodistillation	Water	GC-MS GC-FID	α-Thujene	Monoterpene	4.7% *	[20]
					δ-Terpinene	Monoterpene	3.9% *	
					Methyl eter thymol	Monoterpene	3.8% *	
					Thymol	Monoterpene	5.2% *	
					Carvacrol	Monoterpene	74.1% *	
					β-Caryophylene	Sesquiterpene	5.1% *	
	Seeds	Soxhlet extraction	n-hexane	GC-MS	Palmitic acid	Fatty acid	3.2% *	[48]
					Gadoleic acid	Fatty acid	27.5% *	
					Oleic acid	Fatty acid	9.7% *	
					Linoleic acid	Fatty acid	1.7% *	
					Arachidonic acid	Fatty acid	15.8% *	
					Eicosenoic acid	Fatty acid	69.6% *	
					Saturated fatty acids	Fatty acid	19.0% *	
					Unsaturated fatty acids	Fatty acid	81.0% *	
*S. salzmanniana*	Seeds	Soxhlet extraction	Hexane	GS-MS	Palmitic acid	Fatty acid	1.0% *	[49]
					Arachidic acid	Fatty acid	3.4% *	
					Behemic acid	Fatty acid	3.4 *	
					Oleic acid	Fatty acid	7.6% *	
					Eicosanoid acid	Fatty acid	64.7% *	
					Erucic acid	Fatty acid	19.0% *	
	Stems	No information	Methanol	TLC	Salzmannianoside A	Saponin	Identified	[50]
					Salzmannianoside B	Saponin	Identified	
					Pulsatilla saponin D	Saponin	Identified	
					3-*O*-[[β-D-glucopyranosyl-(1→4)]-[α-L-rhamnopyranosyl-(1→2)]-α-L-arabinopyranosyl]oleanolic acid	Saponin glycoside	Identified	
*S. caracasana*	Seeds	Soxhlet method	Hexane	GC-MS	Palmitic acid	Fatty acid	2.2% *	[49]
					Stearic acid	Fatty acid	1.6% *	
					Arachidic acid	Fatty acid	9.6 *	
					oleic acid	Fatty acid	8.8% *	
					Eicosanoid acid	Fatty acid	69.4% *	
					Erucic acid	Fatty acid	5.0% *	
					Linoleic acid	Fatty acid	1.4% *	
	Aerial parts	Maceration	96% methanol solution	GC–MS NMR	Spathulenol	Sesquiterpene	4.2% *	[39]
					6,10,14-Trimethyl-2-pentadecanone	Terpenoid	7.3% *	
					Methyl palmitate	Fatty acid ester	7.7% *	
					β-Sitosterol	Phytosterol	21.4 mg DM **	
					β-Amyrin	Triterpene	1698. mg DM **	
					Friedelin	Triterpene	5.6 mg DM **	
					Stigmasterol	Phytosterol	37.8 mg DM **	
					β-Sitosterol glucoside	Phytosterol glycoside	3.2 mg DM **	
					Allantoin	Diureide	6.5 mg DM **	
					Quercitrin	Flavonol Glycoside	30.9 mg DM **	
*S. goniocarpa*	Leaves	Maceration	Methanol	IR GC-MS NMR	Goniocarpic acid	Sesterpene	Identified	[21]
					Phytol	Diterpene alcohol	Identified	
*S. schiedeana*	Stems	Maceration	Methanol (5:1 mL/g)	Colorimetric test	Alkaloids	Alkaloids	Identified	[36]
					Flavonoids	Phenolic compounds	Identified	
					Tannins	Polyphenols	Identified	
	Stems	Maceration	Methanol (5:1 mL/g)	Colorimetric test	Alkaloids	Alkaloids	Identified	[37]
					Flavonoids	Phenolic compounds	Identified	
					Tannins	Polyphenols	Identified	
	Stem	Maceration	Methanol (2.5:1 mL/g)	TLC HPLC	epicatechin– (4β → 8)–epicatechin–(4β → 8, 2β →*O*→ 7) epicatechin	Flavanol	Identified	[40]
	Stem	Maceration	Methanol	GC/MS	Phytol	Diterpene alcohol	1.18% *	[51]
					Phytone	Sesquiterpene	1.13 *	
					Methyl palmitate	Fatty acid ester	38.66 *	
					Methyl arachidate	Fatty acid ester	1.05 *	
					4,8,12,16-tetramethylpentadecan-4-olide	Macrolide lactone	15.99 *	
					Methyl linoleate	Fatty acid ester	17.17 *	
					Methyl stearate	Fatty acid ester	1.23 *	
					Methyl behenate	Fatty acid ester	2.07 *	
					Methyl tetradecanoate	Fatty acid ester	12.97 *	
					Tert-butyl (4-(2,6-di-tert-butyl-4-methoxyphenoxy)-3-nitro-4-oxobutyl)prolinate	Phenolic acid	4.13 *	
*S. yucatanensis*	Leaves	Maceration	Ethanol for extraction and fractionation with hexane and ethyl acetate	TLC GC-MS	lup-20(29)-en-3-one	Triterpene	Identified	[22]
					β-caryophyllene oxide	Sesquiterpene	Identified	
*S. triquetra*	Aereal parts	Sequential extractions: n-hexane, methanol and ethyl acetate, evaporated to dryness	No information	IR 1H/13C NMR EIMS	Stigmasterol	Phytosterol	Identified	[7]
					Oleanolic acid	Fatty acid	Identified	
					Morolic acid	Triterpene	Identified	
					Hederagenin	Saponin	Identified	
					11α-hydroperoxy-hederagenin	Saponin	Identified	
	Stems	Maceration (collected extract was concentrated by rotatory evaporator)	85% ethanol solution	OCC TLC UPLC-MS RMN	Ethyl palmitate	Fatty acid ester	25.77% **	[6]
					Stigmasta-3,5-dien-7-one	Steroid	13.92% **	
					Methyl pentacosanoate	Fatty acid ester	10.75% **	
					Ethyl docosanoate	Fatty acid ester	8.64% **	
					Ethyl oleate	Fatty acid ester	4.97% **	
					Stigmasta-5,22-dien-3-ol	Phytosterol	9.96% **	
					Erucic acid	Fatty acid	1.43% **	
*S. racemosa*	Leaves	Maceration (collected extract was filtered and concentrated by rotatory evaporator)	Hexane Ethyl acetate Methanol (50 g/300 mL)	NMR	Saponins	Terpenes	Identified	[12]
					Flavonoids	Glycosylated flavonoids	Identified	

* Relative amount after comparison of the integrative area of the chromatogram; ** Estimated value from the yield extract; DM: dry matter.

**Table 3:** Research reports and toxicological evaluations of *Serjania marginata* extracts.

Activity	Plant Part/Conditioning Sample	Extraction Method/Extract Conditioning	Solvent/Liquid-to-Solid Ratio	Resuspension/ Fractionation	Dose/ Concentration	In Vitro/In Vivo Model	Model Assay/ Control	Relevant Results	Ref.
Antimicrobial	Leaves (Dried at 60 °C/24 h, concentrate, and lyophilized)	Maceration (Extract was concentrated via evaporation)	Water (1:10 mL/mg)	Resuspension with distilled water	15–1000 µg/mL	*Burkholderia cepacia*, *Enterococcus faecalis*, *Escherichia coli*, *Pseudomonas aeruginosa*, *Staphylococcus. epidermidis*, *Staphylococcus aureus*, *Staphylococcus saprophyticus*	Microdilution Control: Tetracycline	All aqueous extracts exhibited antibacterial properties	[5]
Antimicrobial	Leaves	Maceration	Ethanol: water 7:3 *v*/*v*	Resuspended in 0.9% saline solution	7.81–1000 µg/mL	*Escherichia coli*, *Staphylococcus aureus*, *Salmonella setubal*, *Helicobacter pylori*, *Candida albicans*	Microdilution Controls: ampicillin and amoxicillin	The extract showed antimicrobial activity in a concentration- and strain-dependent response	[10]
Antimicrobial	Leaves (Dried at 60 °C)	Maceration (Concentrated via evaporation)	95% ethanol solution	Hexane fraction (rich in essential oils)	0.1–1000 µg/mL	*Bacillus toyonensis*, *Bacillus thuringiensis*, *Bacillus cereus*, *Bacillus proteolyticus*	Disk diffusion/No information	The extract did not show antimicrobial activity against *Bacillus* species	[61]
Antimicrobial	Leaves (Dried at room temperature)	Percolation (Extract was lyophilized)	70% ethanol solution	Resuspended in 5% DMSO solution	0.98–1000 µg/mL	*Mycobacterium tuberculosis*, *Klebsiella pneumoniae*, *Pseudomonas aeruginosa*, *Staphylococcus epidermidis*	Microdilution/No information	The extract exhibited antimicrobial effects in a concentration- and strain-dependent response	[62]
Anti-inflammatory	Leaves	Maceration	Hydroalcoholic	Resuspended in saline solution (0.9%)	30, 100, and 300 mg/kg	Murine model	Carrageenan-induced paw edema	The extract showed anti-inflammatory properties	[11]
Anti-inflammatory	Leaves (Dried at room temperature)	Percolation (Extract was lyophilized)	70% ethanol solution	Resuspended in saline solution (0.9%)	30, 100, and 300 mg/kg	Murine model	Carrageenan-induced paw edema/Dexamethasone	The extract showed anti-inflammatory properties	[62]
Antinociceptive	Leaves	Maceration	Hydroalcoholic (10 g/100 mL)	Resuspended in saline solution (0.9%)	30, 100, and 300 mg/kg	Murine model	Acid formalin-induced nociception	The extracts reduce nociception in a dose-dependent response	[11]
Antihyperalgesic	Leaves	Percolation	Ethanol	Resuspended in saline solution (0.9%)	30, 100, and 300 mg/kg	Swiss murine model	Von Frey acetone test Control: dexamethasone	The extract effectively reduced mechanical hyperalgesia	[11]
Antioxidant	Leaves Steams	Maceration	Water	Lyophilized extract was resuspended in distilled water	50–1000 µg/mL	DPPH	Inhibition of radical	The stems showed higher antioxidant properties than the leaves	[5]
Cytotoxic	Leaves (Dried at room temperature)	Percolation (vacuum concentrated to dryness at 40 °C and lyophilized)	Ethanol (70:30 *v*/*v*)	The dried extract was fractionated with water: butanol solution (30:70 *v*/*v*)	50–500 mg/mL	Non-tumor gastric epithelium cells and gastric adenocarcinoma cells	MTT Negative control: PBS	The extract showed cytotoxicity against cells in a concentration-dependent manner	[26]
Cytotoxic	Leaves	No information	Ethanol	No information	150–300 µg/mL	Human normal and cancer gastric cells	MTT assay Cell proliferation curves AO/EB staining	The extract showed cytotoxicity against cancer (150 µg/mL) and normal cells (µg/mL) in a concentration-dependent manner	[8]
Antimutagenic	Leaves	No information	Ethanol	No information	150–300 µg/mL	Human normal and cancer gastric cells	Cytokinesis-block micronucleus cytome assay	The extract did not show mutagenic effects	[8]
Antimutagenic	Leaves	Maceration	Ethanol: water 7:3 *v*/*v*	Extract diluted in DMSO	2.5–20 mg/plate	*Salmonella typhimurium*	Ames test	It was reported that the absence of mutagenic effects	[10]
Antimutagenic	Leaves (dried at room temperature)	Percolation (vacuum concentrated to dryness at 40 °C and lyophilized)	Ethanol (70:30 *v*/*v*)	The dried extract was fractionated with water: butanol solution (30:70 *v*/*v*)	50–500 mg/mL	Non-tumor gastric epithelium cells and gastric adenocarcinoma cells	MTT:CBMN-cyt Positive control: DXR (0.2 µg/mL)	The extract did not show mutagenic effects	[26]
Hepatoprotective	Leaves (dried at 37 °C for 48 h)	Maceration (lyophilized)	Water	5 mg of dry extract solubilized in minimal methanol	224.3 mg/kg/day	*Nile tilapia*	Feeding trial/Control: commercial food	The extract showed hepatoprotective effects	[27]
Gastroprotective	Leaves	Maceration	Ethanol: water 7:3 *v*/*v*	Resuspended in saline solution (0.9%)	125–500 mg/kg	Wistar rat	Ethanol-induced ulcers, ischemia–reperfusion Controls: carbenoxolone, lansoprazole	The extract reduces the gastric lesions by 60-90% at 500 mg/kg	[10]
Gastroprotective	Leaves (dried at 37 °C for 48 h)	Maceration (lyophilized)	Water	5 mg of dry extract solubilized in minimal methanol	224.3 mg/kg/day	*Nile tilapia*	Feeding trial/Control: commercial food	The extract exhibited gastroprotective effects and stimulated intestinal digestion	[27]
Anti-diarrhea	Leaves	Maceration	Ethanol: water 7:3 *v*/*v*	Resuspended in saline solution (0.9%)	250 mg/kg	Wistar rat	Castor oil-induced diarrhea Control: loperamide and saline solution	The extract did not show a decrease or an increase in the severity of diarrhea	[10]
Photoprotective	Leave Steams	Maceration (vacuum concentrated to dryness at 40 °C and lyophilized	Water	Resuspended in water	200–1000 µg/mL	Spectrophotometer	SPF spectrophotometric method	The leaf extract demonstrated a superior sun protective factor than the steam extract	[5]
Acute oral toxicity	Leaves	Maceration	Ethanol: water 7:3 *v*/*v*	Resuspended in saline solution (0.9%)	5000 mg/kg	Wistar rat	Acute toxicity assay Control: saline solution	No sign and symptoms of toxicity were reported	[10]
Acute and subacute toxicity	Leaves (dried at 37 °C for 48 h)	Maceration (Lyophilized)	Water	Resuspended in saline solution (0.9%)	30–2000 mg/kg	Wistar rats	Subchronic toxicity assay/ Saline solution (0.9%)	No toxicity was observed. However, after 14 days of daily administration, alterations in kidney histology and an increase in abnormal sperm were reported.	[44]
Toxicity	Leaves Steams	Maceration	Water	Resuspended in distilled water	50–1000 µg/mL	*Artemia salina*	Acute toxicity assay Negative control: saline solution	The extract did not show toxicity against *A. salina*	[5]
Insecticide	Leaves (Dried at 40 °C)	Maceration (Evaporation)	Ethanol Water	Resuspended in distilled water	1000–10,000 µg/mL	*Plutella xylostella*	Food preference and oviposition	The extracts were effective as oviposition suppressants for this insect	[63]
Insecticide	Leaves (dried at 40 °C in an air oven)	Maceration	Water	Direct use of filtered extract	5 and 10% *w*/*v*	*Plutella xylostella*	Direct contact toxicity assay on a model	Both concentrations were toxic to eggs and pupae of *P. xylostella*. The 10% concentration was the most effective in terms of larval mortality	[32]
Antiparasitic	Leaves (dried at 40 °C in an air oven)	Maceration	Water	Direct use of filtered extract	5 and 10% *w*/*v*	*Tetrastichus howardi*	Observational assay on morphological changes in the model	The extracts did not show antiparasitic properties against *T. howardi*	[28]

**Table 4:** Research reports and toxicological evaluations of *Serjania erecta* extracts.

Activity	Plant Part/Conditioning Sample	Extraction Method/Extract Conditioning	Solvent/Liquid-to-Solid Ratio	Resuspension/ Fractionation	Dose/ Concentration	In Vitro/In Vivo Model	Model Assay/ Controls	Relevant Results	Ref.
Antimicrobial	Leaves (Dried at 40 °C)	Maceration (Evaporation)	70% ethanol solution	Resuspended in 10% DMSO solution	6.25–50 µg/mL	*Mycoplasma hominis*, *Ureaplasma urealyticum*, *Mycoplasma arginini*	Microdilution Control: DMSO (10%)	The extracts showed antimicrobial activity in concentration- and strain-dependent response	[65]
Antimicrobial	Leaves Roots	Maceration at room temperature	Ethanol	Fractionation: (water, methanol, acetone)	10–400 µg/mL	*Staphylococcus aureus*, *Pseudomonas aeruginosa*, *Escherichia coli*, *Salmonella setubal*, *Saccharomyces cerevisiae*, *Candida albicans*	Rezasurin Microtiter Assay/Control: isoniazis	Leaves and roots inhibited the growth of all tested microorganisms	[17]
Antimicrobial	Leaves (Dried at 60 °C)	Maceration (Evaporation)	Ethanol (95%): water 1:1	Hexane fraction (rich in essential oils)	1–100 µg/mL	*Bacillus toyonensis*, *Bacillus thuringiensis*, *Bacillus cereus*, *Bacillus proteolyticus*	Disk diffusion	The extract showed antimicrobial activity against the tested microorganisms	[61]
Antiparasitic	Leaves (dried at 40 °C in an air oven)	Maceration	Water	Direct use of filtered extract	5 and 10% *w*/*v*	*Tetrastichus howardi*	Direct contact assay Negative control: water Positive control: acephate	The aqueous extract did not interfere with the parasitism of *T. howardi* on 4th instar larvae of *P. xylostella*.	[28]
Anti-inflammatory	Leaves	Sequential extraction	Ethanol, N-hexane, ethyl acetate, ethanol	Fractionated with water, methanol, and acetone	30, 100, and 300 mg/kg	Murine model	Carrageenan pleurisy Zymosan peritonitis	The extract exhibited anti-inflammatory properties in a concentration dependence	[4]
Anti-inflammatory	Steam Leaves	Maceration	Ethanol		0.003–4 mg/ear	Murine model	Ear edema	Topical application of the extract and its fractions caused a dose-dependent reduction in ear edema and tissue myeloperoxidase activity	[16]
Anti-inflammatory	Leaves (Dried)	Maceration (lyophilized)	95% Ethanol	Saline solution	300 mg/kg	Murine model	Induction of inflammatory pulp tissue/Saline solution	The extract did not show anti-inflammatory effects on pulp tissue in the analyzed periods.	[66]
Antioxidant	Leaves Steam Roots	Decoction 24 h in the dark	Distilled boiling water (90 g/900 mL)	Crude extract	2.5–10 mg/mL	DPPH	Inhibition of radical/Rutin as a positive control	The extracts exhibited antioxidant properties	[13]
Antioxidant	Leaves Roots	Maceration at room temperature	Ethanol	Fractionation: with water, methanol, acetone	10–400 µg/mL	DPPH β-carotene–linoleic acid assay	Inhibition of radical/Quercetin β-carotene-linoleic acid assay/BHT	The extracts showed low antioxidant properties	[17]
Antioxidant	Leaves	Maceration (Evaporation)	Methanol Chloroform	No information	40–640 mg/mL	DPPH β-carotene–linoleic acid assay	DPPH test Positive control: quercetin β-carotene–linoleic acid assay Positive control: quercetin	The extract showed antioxidant properties	[45]
Analgesic	Leaves	Direct extraction Sequential extraction (concentrated under vacuum)	Ethanol N- hexane, ethyl acetate, ethanol	Fractionation with water, methanol, and acetone	30, 100, and 300 mg/kg	Mice Leukocytes Neutrophils	Formalin test control/ Control indomethacin, morphine	The extract showed analgesic properties in a dose-dependent manner	[4]
Neuroprotective	Leaves	Maceration for three days	Methanol (concentrated under vacuum at 50 °C and further lyophilized)	The crude extract was dissolved in methanol	25–200 µg/mL	Rat adrenal pheochromocytoma (PC12) cell line	MTT assay (cell viability), controls: untreated cells and Aβ-treated cells	Isolated compounds from leaves of *S. erecta* exhibited neuroprotective effects in a concentration-dependent response	[14]
Anti-hypertensive	Roots	Decoction	Water	Administered with condensed milk (oral intake)	10 mL of 5% *w*/*v* solution	Murine model	Direct measurement of blood pressure	The treatment improved endothelial function and promoted nitric oxide production by the endothelium	[15]
Anti-ulcer	Leaves	Maceration in chloroform and methanol Three extractions, 48 h each: concentrated by rotary evaporation at 38 °C	Extract in chloroform: (4 L/Kg) Extract in methanol: (4 L/Kg)	No information	125–5000 mg/kg	In vivo Murine model	Ethanol-induced gastric ulcer model/Control positive: carbenoxolone: 100 mg/kg	The extract showed gastroprotective properties	[45]
Antivenom	Leaves (dried at 60 °C in an air oven)	Maceration for 72 h (concentrated via rotary evaporation at 50 °C)	Methanol	Extract was fractionated	Venom/toxin with the extracts at a 1:30 (*w*/*w*)	Enzymatic assay	Indirect hemolytic assay for phospholipase A_2_ activity. Positive control: *Bothrops jararacussu* snake venom Negative control: PBS	The crude extract and the fractions neutralized the toxic activities of the *Bothrops jararacussu* snake venom and the isolated myotoxins.	[29]
Toxicity	Leaves Steam Roots	Decoction 24 h in the dark	Distilled boiling water (90 g/900 mL)	Crude extract	50–1250 mg/kg	Murine model	Screening, biochemical and hematological analysis	The extract did not show toxicity	[13]
Toxicity	Leaves	Maceration in chloroform and methanol Three extractions, 48 h each: concentrated by rotary evaporation at 38 °C	Extract in chloroform: (4:1 L/Kg) Extract in methanol: (4L/Kg)	No information	125–5000 mg/kg	Murine model	Acute oral toxicity assay Control group: saline solution	The extract did not promote acute oral toxicity	[45]
Toxicity	Leaves	Maceration: for 24 h at room temperature, followed by filtration and lyophilization	Water 1:1000 mL/µg	Water at different concentrations 2.5–150 µg/mL	2.5–150 µg/mL	*Piaractus mesopotamicus*	Toxicity bioassay: Blood plasma biochemical analysis and electrolyte assay Histopathological analysis: Light microscopy-based histopathology/untreated fish Morphometric analysis: organ morphometric/untreated fish	The extract caused morphofunctional and histological alterations in the gills and liver. Mortality occurred at extract levels above 50 μg/mL	[43]
Insecticide	Leaves (dried at 40 °C in an air oven)	Maceration	Water	Direct use of filtered extract	5 and 10% *w*/*v*	*Plutella xylostella*	Direct contact	Both concentrations were toxic to eggs, larvae, and pupae of *P. xylostella*	[28]
Pesticide	Leaves (dried at 40 °C/120 h)	Maceration (Evaporation)	Methanol	Resuspended in distilled water containing 2.5% (*v*/*v*) methyl alcohol	0.0078–20 µg/mL	*Chrysodeixis includens*	Inhibition of growth and development Control: distilled water containing 2.5% (*v*/*v*) methyl alcohol	Extract treatments increased the duration of the larval, pupal, and total development	[67]

## Data Availability

No new data were created or analyzed in this study. Data sharing is not applicable to this article.

## Supplementary Materials

The following supporting information can be downloaded at: https://www.mdpi.com/article/10.3390/molecules31091477/s1, *Serjania* species information.

## Abbreviations

The following abbreviations are used in this manuscript:

DNA	Deoxyribonucleic acid
TLC	Thin-layer chromatography
HPLC	High-Performance Liquid Chromatography
FIA-ESI-IT-MS	Flow Injection Analysis–Electrospray Ionization–Ion Trap–Mass Spectrometry
UHPLC-(ESI)-HRMS	Ultra-High Performance Liquid Chromatography-Electrospray Ionization–High-Resolution Mass Spectrometry
NMR	Nuclear Magnetic Resonance
GS-MS	Gas Chromatography–Mass Spectrometry
GS-FID	Gas Chromatography Flame Ionization Detection
HPLC-PDA	High-Performance Liquid Chromatography with Photodiode Array Detection
IR	Infrared spectroscopy
OCC	Open-Column Chromatography
UPLC-MS	Ultra-Performance Liquid Chromatography-Mass Spectrometry
FTIR	Fourier Transform Infrared
EI-MS	Electron Ionization Mass Spectrometry
COX	Cyclooxygenase
LOX	Lipoxygenase
DMSO	Dimethyl sulfoxide
HPLC-DAD	High-Performance Liquid Chromatography with Diode Array Detection
DPPH	2,2-diphenyl-1-picrylhydrazyl
ABTS	2,2′-azino-bis(3-ethylbenzothiazoline-6-sulfonic acid)
MIC	Minimum Inhibitory Concentration
MTT	3-[4,5-dimethylthiazol-2-yl]-2,5 diphenyl tetrazolium bromide
