# Supplementary material for: Traditional Uses, Phytochemicals, Biological Activities, and Biotechnological Applications of Serjania Species: A Review of Current Knowledge and Future Prospects
Source: Molecules. 2026 Apr 29;31(9):1477. doi: 10.3390/molecules31091477 (PMC13165125; doi:10.3390/molecules31091477)

# Supplementary Materials

Important information in Figure 1. Images were obtained from iNaturalist.org, a social network, community, and citizen-science platform dedicated to documenting and identifying biodiversity worldwide (https://www.inaturalist.org/taxa/156116-Serjania/browse_photos). On this website, there are images available for free use under the CC BY and CC BY-NC 4.0 licenses. Thus, the selected images are under these licenses' terms. Detailed information about images and their collaborators are described as follows:

*Serjania erecta*, available at https://www.inaturalist.org/photos/569210987, accessed April 20, 2026.

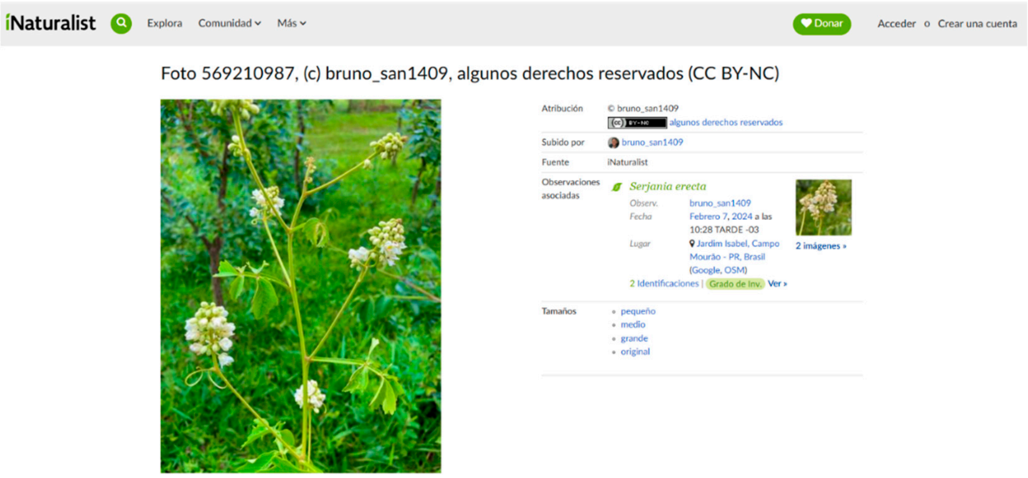


*Serjania lethalis*, https://www.inaturalist.org/photos/301205744, accessed April 20, 2026.

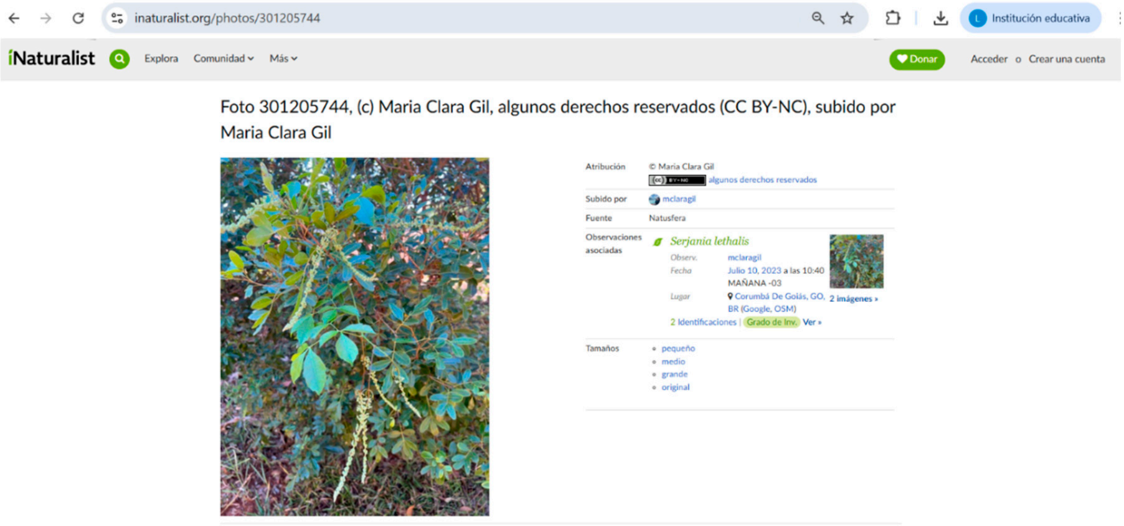

*Serjania salzmaniana*, https://www.inaturalist.org/photos/260572371, accessed April 20, 2026.

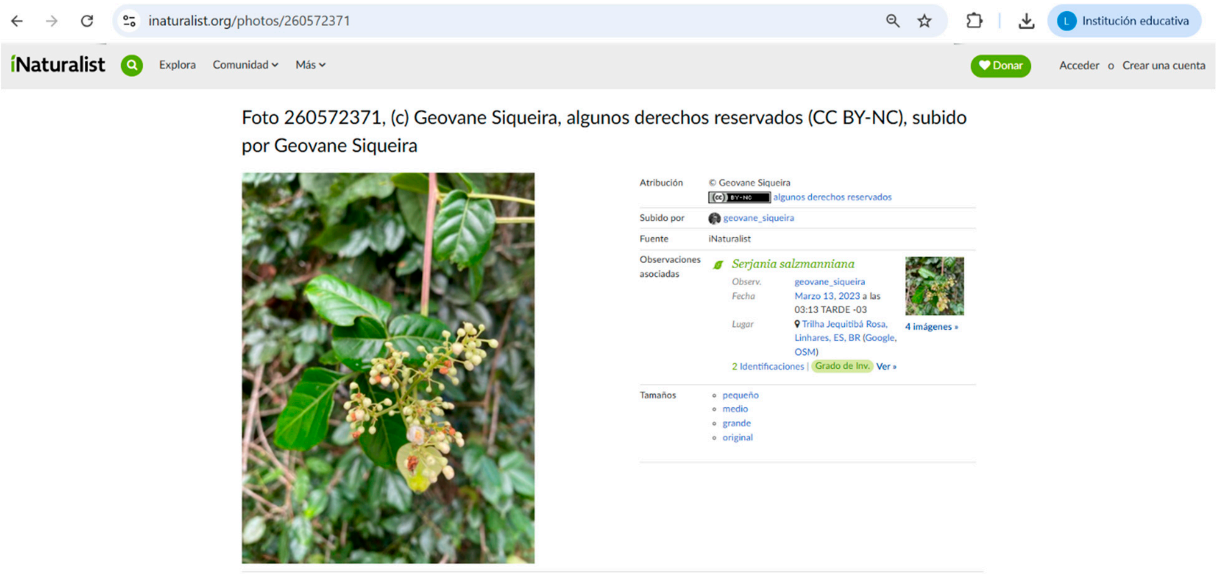


*Serjania glabrata*, https://www.inaturalist.org/photos/59325003, accessed April 20, 2026.

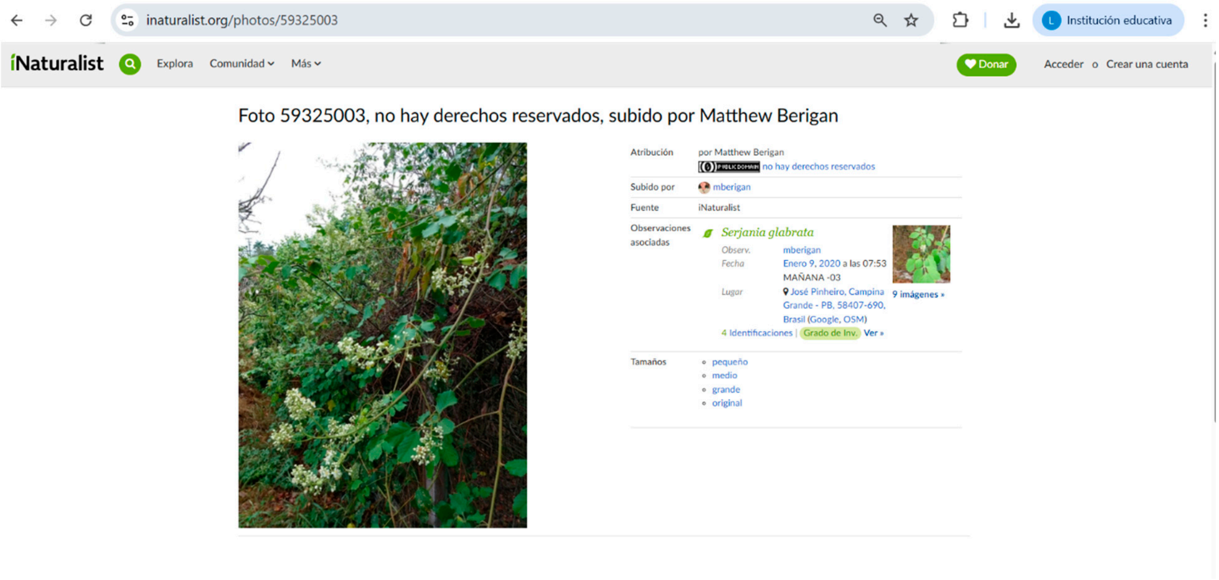

*Serjania triquetra*, https://www.inaturalist.org/photos/169889459, accessed April 20, 2026.

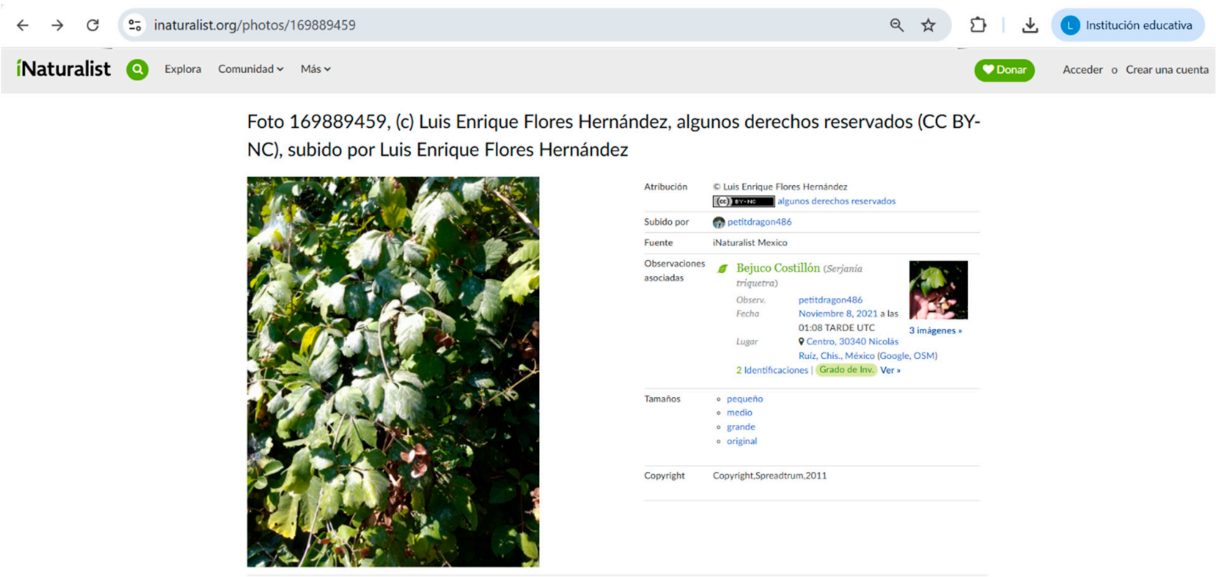


*Serjania marginata*, https://www.inaturalist.org/photos/176475818, accessed April 20, 2026.

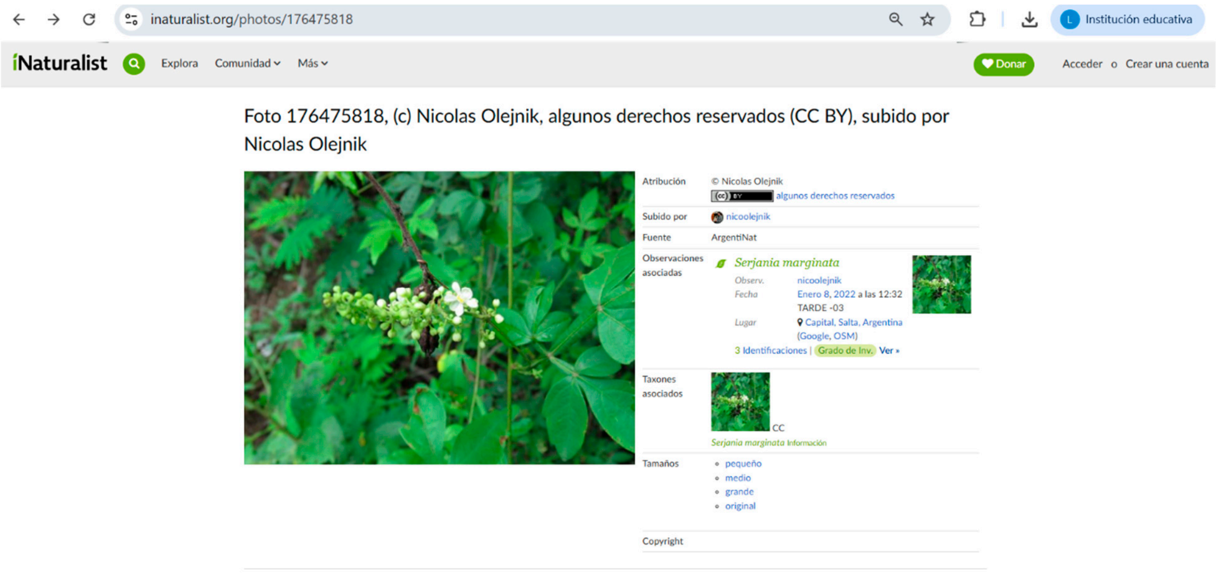

According to the information found (https://creativecommons.org/licenses/by-nc/4.0/ and https://creativecommons.org/licenses/by/4.0/), any person is free to "Share"—copy and redistribute the material in any medium or format, or "Adapt"—remix, transform, and build upon the material. The licensor cannot revoke these freedoms as long as you follow the license terms.

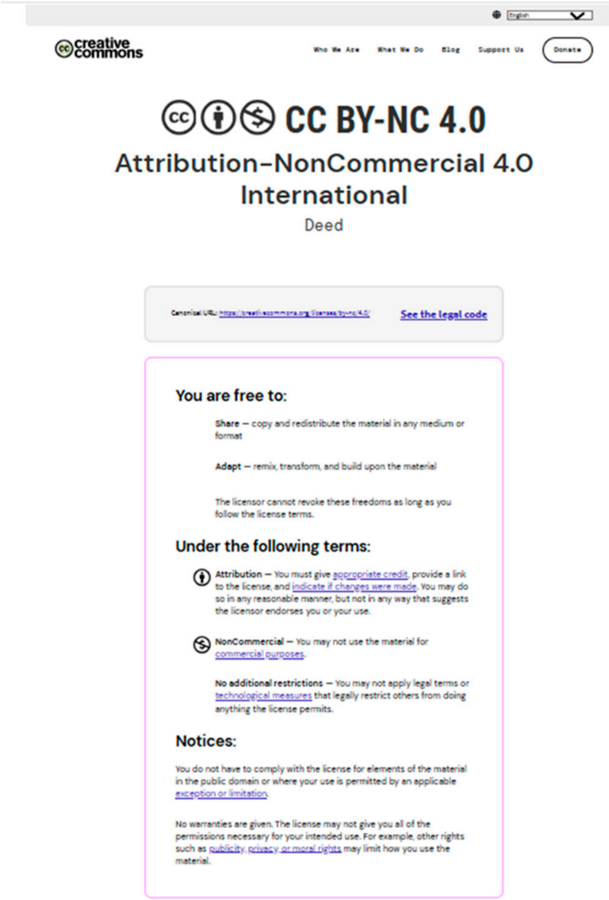


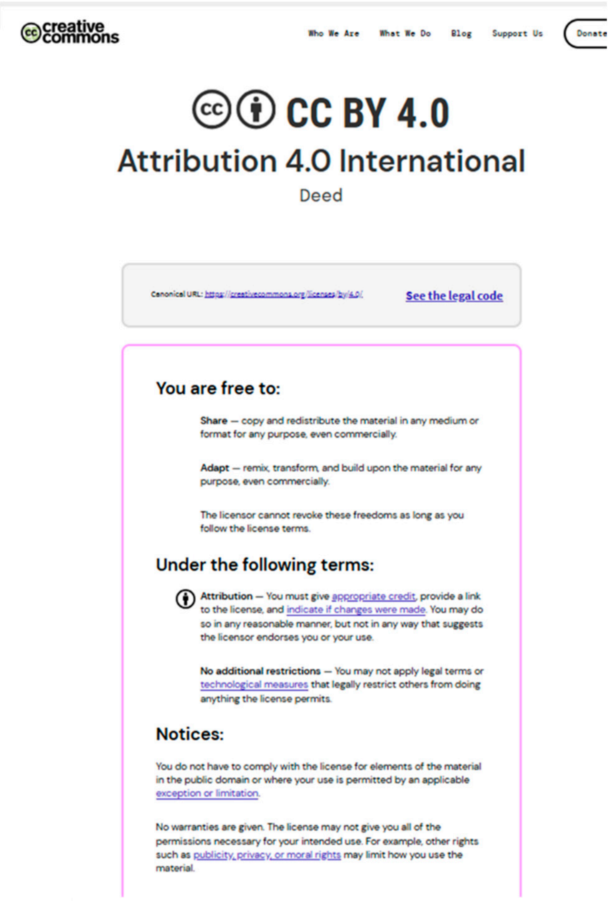

Supplement: Supplementary file 1 [file molecules-31-01477-s001.zip › molecules-4237373-supplementary.pdf]
